# Contrasting Regional Carbon Cycle Responses to Seasonal Climate Anomalies Across the East‐West Divide of Temperate North America

**DOI:** 10.1029/2020GB006598

**Published:** 2020-11-13

**Authors:** B. Byrne, J. Liu, A. A. Bloom, K. W. Bowman, Z. Butterfield, J. Joiner, T. F. Keenan, G. Keppel‐Aleks, N. C. Parazoo, Y. Yin

**Affiliations:** ^1^ Jet Propulsion Laboratory California Institute of Technology Pasadena CA USA; ^2^ Division of Geological and Planetary Sciences California Institute of Technology Pasadena CA USA; ^3^ Joint Institute for Regional Earth System Science and Engineering University of California Los Angeles USA; ^4^ Department of Climate and Space Sciences and Engineering University of Michigan Ann Arbor MI USA; ^5^ Laboratory of Atmospheric Chemistry and Dynamics NASA Goddard Space Flight Center Greenbelt MD USA; ^6^ Earth and Environmental Sciences Area Lawrence Berkeley National Laboratory Berkeley CA USA; ^7^ Department of Environmental Science, Policy and Management University of California Berkeley CA USA

**Keywords:** carbon cycle, interannual variability, North America, terrestrial biosphere model, net ecosystem exchange, gross primary production

## Abstract

Across temperate North America, interannual variability (IAV) in gross primary production (GPP) and net ecosystem exchange (NEE) and their relationship with environmental drivers are poorly understood. Here, we examine IAV in GPP and NEE and their relationship to environmental drivers using two state‐of‐the‐science flux products: NEE constrained by surface and space‐based atmospheric CO_2_ measurements over 2010–2015 and satellite up‐scaled GPP from FluxSat over 2001–2017. We show that the arid western half of temperate North America provides a larger contribution to IAV in GPP (104% of east) and NEE (127% of east) than the eastern half, in spite of smaller magnitude of annual mean GPP and NEE. This occurs because anomalies in western ecosystems are temporally coherent across the growing season leading to an amplification of GPP and NEE. In contrast, IAV in GPP and NEE in eastern ecosystems is dominated by seasonal compensation effects, associated with opposite responses to temperature anomalies in spring and summer. Terrestrial biosphere models in the MsTMIP ensemble generally capture these differences between eastern and western temperate North America, although there is considerable spread between models.

## Key Points

- GPP and NEE IAV in western temperate North America is characterized by amplification, with enhance uptake in cooler‐wetter conditions
- GPP and NEE IAV in eastern temperate North America is characterized by compensating anomalies between spring and summer
- The MsTMIP models generally capture these east‐west differences in NEE and GPP IAV

## 1 Introduction

Interannual variations (IAVs) in climate are a major driver of IAV in gross primary productivity (GPP) and net ecosystem exchange (NEE). Understanding the relationship between ecosystems and climate variability is important for predicting the response of ecosystems to climate variability, such as droughts and heatwaves, as well as the response of ecosystems to climate change (Baldocchi, Ryu, et al., 2016; Cox et al., 2013; Niu et al., 2017). However, the mechanisms underlying the responses of ecosystems to climate variability are still not well understood and vary between ecosystems (Baldocchi et al., 2018; Niu et al., 2017).

A long‐standing challenge in carbon cycle science has been to study IAV in GPP and NEE on large subcontinental spatial scales (approximately thousands of km). Estimating fluxes on these scales from “bottom‐up” estimates of ecosystem function based of site level experiments is challenging due to spatial heterogeneity. Conversely, top‐down estimates of NEE obtained through observations of atmospheric CO_2_ have generally only provided constraints on CO_2_ fluxes on the largest (continental‐to‐global) scales, due to sparsity of observations.

Recently, space‐based measurements of column‐averaged dry‐air mole fractions of CO_2_ ( $X_{{\text{CO}}_{2}}$) have allowed for much expanded observational of coverage, leading to top‐down NEE constraints on smaller spatial scales (Bowman et al., 2017; Byrne et al., 2017, 2019, 2020; Guerlet et al., 2013; Ishizawa et al., 2016; Liu et al., 2017; Liu, Bowman, Parazoo, et al., 2018). Furthermore, advances in remote sensing techniques have allowed for more reliable GPP estimates from space from solar‐induced fluorescence (SIF) measurements (Byrne et al., 2018; Frankenberg et al., 2011; Joiner et al., 2011; Parazoo et al., 2014; Sun et al., 2017; Yang et al., 2015) and up‐scaled flux tower GPP estimates using MODIS observations (Joiner et al., 2018; Jung et al., 2020).

In this study, we examine the ability of two novel CO_2_ flux constraints to recover IAV in GPP and NEE on subcontinental scales within temperate North America. We employ state‐of‐the‐science observationally constrained GPP and NEE products for examining IAV. The FluxSat GPP product (Joiner et al., 2018) is based on an MODIS remote sensing calibrated against global eddy covariance flux measurements and has been found to produce more realistic IAV in GPP when compared to FLUXNET sites relative to other upscaled GPP products (Joiner et al., 2018). The flux inversion NEE product used here is reported in Byrne et al. (2020). This product is derived from a global CO_2_ flux inversions and is unique in that it assimilates both surface‐ and space‐based CO_2_ measurements, providing increased observational constraints relative to single data set NEE flux inversion products.

For this analysis we focus on temperate North America, which we have chosen for two reasons. First, temperate North America is comparatively well sampled by both eddy covariance sites (which are used to calibrate FluxSat GPP estimates) and surface‐based CO_2_ measurements (which are assimilated in the NEE flux inversions). Second, temperate North America has a substantial east‐west gradient in moisture. Much of western temperate North America (particularly the southwest) is characterized by moisture‐limited ecosystems, while the east is less moisture limited and has many forest and cropland ecosystems. These different ecosystems types likely have differences in their responses to climate variability.

Globally, moisture‐limited ecosystems have been shown to play an out‐sized role in internnual variability (IAV) of the atmospheric CO_2_ growth rate (Ahlström et al., 2015; Fu et al., 2017; Huang et al., 2016; Poulter et al., 2014), relative to what would be expected given their productivity. The reason that these ecosystem experience such large IAV in CO_2_ net uptake is thought to be linked to moisture availability (Huang et al., 2016). In these ecosystems, negative GPP anomalies are driven by warm‐dry conditions, and positive GPP anomalies are driven by cool‐wet conditions (Ahlström et al., 2015). In turn, NEE anomalies in these ecosystems are strongly associated with variations in GPP (Ahlström et al., 2015). Consistent with these large‐scale analyses, site level observations of moisture‐limited ecosystems in southwestern North America have shown strong sensitivity to water availability for GPP and NEE (Biederman et al., 2016, 2018). Still, the relative impact of these ecosystems on temperate North American carbon fluxes is not well characterized.

IAV in eastern temperate North American ecosystems has been shown to have seasonally compensating effects, defined as temporally anticorrelated anomalies during a growing season. For example, a number of studies have found that enhanced GPP early in the growing season is associated with reduced GPP later in the growing season over midlatitude cropland and forest ecosystems (Buermann et al., 2013, 2018; Butterfield et al., 2020; Wolf et al., 2016). There are several possible mechanisms for explaining seasonal compensation effects. Enhanced spring GPP is associated with warmer spring temperatures (Angert et al., 2005; Wolf et al., 2016). Warmer temperatures early in the growing season result in increased evapotranspiration leading to reduced soil moisture later in the growing season, which adversely impacts productivity (Liu, Kimball, et al., 2020; Parida & Buermann, 2014; Wolf et al., 2016). Direct phenological mechanisms may also contribute to seasonal compensation effects, as the timing of spring budburst and autumn senescence has been found to be correlated on the scale of individual organisms and the landscape (Fu et al., 2014; Keenan & Richardson, 2015). The impact of seasonal compensation effects on annual GPP anomalies has been studied across northern forests and croplands using upscaled FLUXNET GPP (Buermann et al., 2013), Normalized difference vegetation index (NDVI) (Buermann et al., 2018), and SIF (Butterfield et al., 2020), while seasonal compensation in NEE has been examined for the 2011 Texas‐Mexico drought (Liu, Bowman, Parazoo, et al., 2018), 2012 temperate North America drought (Liu, Bowman, Parazoo, et al., 2018; Wolf et al., 2016), and 2018 MidWest floods (Yin et al., 2020). However, the implications of seasonal compensation effects on variability in the carbon balance across multiple years over temperate North America have not yet been examined.

Using the 6 years of NEE estimates from Byrne et al. (2020) in combination with 17 years (2001–2017) GPP from FluxSat, we examine the importance of seasonal compensation effects in GPP and NEE across North America. First, we characterize the extent to which seasonal compensation effects impact growing season GPP and NEE anomalies across North America, and their dependence on temperature and moisture anomalies. Then, we examine the relative contribution of eastern and western North America to the mean seasonal cycle and IAV of GPP and NEE for temperate North America as a whole and compare our data‐driven estimates to modeled fluxes from the Multi‐scale Synthesis and Terrestrial Model Intercomparison Project (MsTMIP).

This paper is organized as follows. Section 2 describes the data sets used in this study, and section 3 describes the methods. Section 4 describes the results: We first describe the dominant modes of IAV recovered the FluxSat GPP and flux inversion NEE (section 4.1) and then examine the consistency of these results with independent CO_2_ flux estimates (section 4.2). Section 4.3 examines the relationship between IAV in ecosystem CO_2_ fluxes with IAV in environmental variables, and section 4.4 examines the implication of east‐west differences in IAV for the North American carbon cycle and the ability of the MsTMIP ensemble to reproduce these differences. Section 5 provides a discussion of the results found in this study, with section 5.1 discussing possible mechanisms explaining east‐west differences in IAV and section 5.2 presenting the implications for the temperate North American carbon sink. Finally, section 6 presents the conclusions.

## 2 Data

We utilize a number of CO_2_ flux data sets to examine IAV in GPP and NEE over temperate North America, as well as environmental data to examine the relationship between CO_2_ fluxes and climate variability. Table 1 gives a list of data sets used in this study, with some additional details provided in this section and in the supporting information.

**Table 1:** Table of Data Sets Used in This Study

Dataset	Time period	Spatial resolution/vegetation type	Reference
GPP and related products (section 2.1)			
FluxSat	2001–2017	0.5° × 0.5°	Joiner et al. (2018)
GOME‐2 SIF	2007–2015	0.5° × 0.5°	Joiner et al. (2016)
NDVI	2001–2015	1.0° × 1.0°	Huete et al. (2002)
FLUXCOM	2000–2013	0.5° × 0.5°	Tramontana et al. (2016)
Flux inversion NEE (section 2.2)			
Byrne et al.	2010–2015	4.0° × 5.0°	Byrne et al. (2020)
CT2017	2000–2016	1.0° × 1.0°	Peters et al. (2007)
CT‐L	2007–2015	1.0° × 1.0°	Hu et al. (2019)
CAMS	2000–2018	1.875° × 3.75°	Chevallier et al. (2010)
Model CO_2_ fluxes (section 2.3)			
MsTMIP	1980–2010	0.5° × 0.5°	Huntzinger, Schwalm, et al. (2018)
Environmental Data (section 2.4)			
Soil Temperature	2001–2017	50 km × 50 km	Reichle et al. (2017)
ESA CCI	2001–2017	0.25° × 0.25°	Liu et al. (2012, 2011)
GPCP	2001–2017	2.5° × 2.5°	Adler et al. (2003)
GRACE TWS	2010–2014	1.0° × 1.0°	Tapley et al. (2004)
FLUXNET sites			
US‐ARM	2003–2012	Croplands	Biraud et al. (2016)
US‐Blo	1997–2007	Evergreen Needleleaf Forests	Goldstein (2016)
US‐GLE	2005–2014	Evergreen Needleleaf Forests	Massman (2016)
US‐Los	2000–2010, 2014	Permanent Wetlands	Desai (2016c)
US‐MMS	1999–2014	Deciduous Broadleaf Forests	Novick and Phillips (2016)
US‐Ne1	2002–2013	Croplands	Suyker (2016a)
US‐Ne2	2002–2013	Croplands	Suyker (2016b)
US‐Ne3	2002–2013	Croplands	Suyker (2016c)
US‐NR1	1999–2014	Evergreen Needleleaf Forests	Blanken et al. (2016)
US‐PFa	1996–2014	Mixed Forests	Desai (2016a)
US‐SRG	2008–2014	Grasslands	Scott (2016d)
US‐SRM	2004–2014	Woody Savannas	Scott (2016a)
US‐Ton	2001‐2014	Woody Savannas	Baldocchi and Ma (2016)
US‐UMB	2000–2014	Deciduous Broadleaf Forests	Gough et al. (2016a)
US‐UMd	2007–2014	Deciduous Broadleaf Forests	Gough et al. (2016b)
US‐Var	2000–2014	Grasslands	Baldocchi, Ma, et al. (2016)
US‐WCr	1999–2006, 2010–2014	Deciduous Broadleaf Forests	Desai (2016b)
US‐Whs	2007–2014	Open Shrublands	Scott (2016c)
US‐Wkg	2004–2014	Grasslands	Scott (2016b)

*Note*. Time period indicates time range examined in this study. The spatial resolution of the data sets are given for gridded data and the vegetation type if given for FLUXNET sites. All gridded data sets are regridded from the listed spatial resolution to 4° × 5° by area weighting.

### 2.1 GPP and Related Products

To examine IAV in GPP, we employ the FluxSat GPP product. We also examine the robustness of these results through comparison with Global Ozone Monitoring Experiment‐2 (GOME‐2) SIF, Moderate Resolution Imaging Spectroradiometer (MODIS) NDVI, and FLUXCOM upscaled GPP estimates.

FluxSat Version 1 (Joiner et al., 2018) estimates GPP based primarily on Nadir BRDF‐Adjusted Reflectances (NBAR) from the MODerate‐resolution Imaging Spectroradiometer (MODIS) MYD43D product (Schaaf et al., 2002) that uses data from MODIS instruments on National Aeronautics and Space Administration (NASA) Aqua and Terra satellites. The GPP estimates are calibrated with the FLUXNET 2015 GPP derived from eddy covariance flux measurements at Tier 1 sites (Baldocchi et al., 2001). The data set also employs SIF from the Global Ozone Monitoring Experiment 2 (GOME‐2) on the EUMETSAT MetOp‐A satellite to identify regions of high productivity crops. FluxSat was evaluated by comparison with independent flux measurements (i.e., not used in the training) and compared very well both in terms of IAV and site‐to‐site variability.

For comparison with SIF, we use the GOME‐2 Version 28 (V28) 740 nm terrestrial SIF data (Joiner et al., 2013, 2016). SIF is the emission of radiation by chlorophyll during photosynthesis and thus provides a proxy for GPP (Papageorgiou & Govindjee, 2007). A “daily correction” is performed to estimate daily average SIF from the instantaneous measurements.

We examine MODIS NDVI over the period 2001–2015. We downloaded MODIS/Terra Monthly Vegetation Indices Global 1x1 degree V005 (MODVI) data set from Earthdata (https://earthdata.nasa.gov). The global monthly gridded MODIS vegetation indices product is derived from the standard 0.05 CMG MODIS Terra Vegetation Indices Monthly product MOD13C2 (Huete et al., 2002) Collection 5.

FLUXCOM RS + METEO products are generated using upscaling approaches based on machine learning methods that integrate FLUXNET site level observations, satellite remote sensing, and meteorological data (Jung et al., 2017, 2020; Tramontana et al., 2016) to generate gridded 0.5° × 0.5° daily CO_2_ flux estimates. Up‐scaled GPP is calculated using three different machine learning algorithms: random forests (RF), multivariate regression splines (MARS), and an artificial neural network (ANN). In this study we examine RF GPP, MARS GPP, and ANN GPP regridded to 4° × 5° and monthly values.

### 2.2 Flux Inversion NEE

To examine IAV in NEE, we employ the combined “GOSAT + surface + TCCON” product of Byrne et al. (2020). This product is unique in that it assimilates both surface‐ and space‐based CO_2_ measurements, providing increased observational constraints relative to other top‐down NEE flux inversion products. We examine the robustness of these results through comparison with three independent CO_2_ flux inversion products that assimilate only flask and in situ CO_2_ observations: CarbonTracker 2017 (CT2017) (Peters et al., 2007, with updates documented at https://www.esrl.noaa.gov/gmd/ccgg/carbontracker/), CarbonTracker Lagrange (CT‐L) (Hu et al., 2019), and Copernicus Atmosphere Monitoring Service (CAMS) greenhouse gases inversion v18r3 (Chevallier, 2013; Chevallier et al., 2005, 2010; Remaud et al., 2018) (downloaded from https://atmosphere.copernicus.eu/). Detailed descriptions of these flux inversions are provided in the supporting information (Text S1).

The NEE fluxes of Byrne et al. (2020) are produced from a flux inversion analyses spanning 2010–2015. The flux inversions assimilate CO_2_ measurements from the Greenhouse Gases Observing Satellite (GOSAT), Total Carbon Column Observing Network (TCCON), and the surface in situ and flask measurements network concurrently. Four‐dimensional variational (4‐DVar) assimilation was implemented to estimate 14‐day scaling factors for prior NEE and ocean fluxes at 4° × 5° spatial resolution using the Greenhouse gas framework—Flux model (GHGF‐Flux). The optimized fluxes are taken to be the average of three flux inversions that employ different prior NEE fluxes and errors. These three flux inversions employ prior fluxes from the simple biosphere model (SiB3), the Carnegie‐Ames‐Stanford Approach (CASA) model, or FLUXCOM. Posterior NEE fluxes are aggregated to monthly mean values for this analysis. A detailed description of the experimental setup and evaluation of the fluxes can be found in Byrne et al. (2020). We also contrast the posterior IAV of the “GOSAT + surface + TCCON” ensemble of inversions with the flux inversions assimilating only surface‐based flask and in situ meansurements, referred to as “surface only”. These data were downloaded online (from https://cmsflux.jpl.nasa.gov/).

### 2.3 MsTMIP Models

MsTMIP is a model intercomparison experiment conducted by the temperate North American Carbon Program (Huntzinger et al., 2013; Wei et al., 2014). The project is designed to provide a consistent and unified modeling framework in order to isolate, interpret, and address differences in process parameterizations among TBMs. In this analysis, we examine the modeled NEE (defined here as MsTMIP NEP × −1) and GPP from the MsTMIP Version 1 SG3 simulation, in which the models are driven by CRU + NCEP reanalysis on a global 0.5° × 0.5° spatial grid with time‐varying land‐use history and atmospheric CO_2_, but with nitrogen deposition kept constant. We examine modeled fluxes over the period 1980–2010. These data were downloaded from the ORNL DAAC (Huntzinger, Schwalm, et al., 2018).

### 2.4 Environmental Data

Anomalies in CO_2_ fluxes are compared with anomalies in environmental variables that are expected to drive carbon cycle anomalies. In particular, we focus our analysis on the relationship between anomalies in CO_2_ fluxes with anomalies in soil temperature and soil moisture.

Soil temperatures are from the MERRA‐2 (Gelaro et al., 2017; Reichle et al., 2011, 2017) reanalysis. We average the soil temperature over Levels 1–3 (TSOIL1, TSOIL2, and TSOIL3), which reaches a depth of 0.73 m. These data were downloaded from the Goddard Earth Sciences Data and Information Services Center at monthly temporal resolution and 4° × 5° spatial resolution (regridded from model horizontal resolution of ∼50 km).

The ESA CCI‐combined surface soil moisture product (Liu et al., 2011, 2012) was downloaded online (from https://www.esa‐soilmoisture‐cci.org/). We use the combined active and passive soil moisture product. Additional data sets are used for supplemental analysis of the relationship between carbon fluxes and moisture stress. We obtained precipitation estimates from the Global Precipitation Climatology Project (GPCP) Monthly Analysis Product. We use GPCP Version 2.3 Combined Precipitation Dataset (Adler et al., 2003). We also use RL06 monthly mass grids of terrestrial water storage (TWS) anomalies derived from the Gravity Recovery and Climate Experiment (GRACE) mission (Flechtner et al., 2014; Landerer & Swenson, 2012; Tapley et al., 2004).

### 2.5 FLUXNET

The FLUXNET network consists of a number of towers across the globe measuring trace gas concentrations and micrometeorological variables. From these data, the eddy covariance method is applied to estimate fluxes of energy and trace gases between the surface and atmosphere. In this study, we utilize monthly GPP and NEE estimates from a number of FLUXNET2015 sites (Pastorello et al., 2020). For GPP estimates we average together the nightime and daytime partitioning estimates. In this study, we examine FLUXNET sites over temperate North America with six or more full years of observations. This includes the following sites: ARM Southern Great Plains site‐Lamont (US‐ARM), Blodgett Forest (US‐Blo), Glacier Lakes Ecosystem Experiments Site (US‐GLE), Lost Creek (US‐Los), Morgan Monrow State Forest (US‐MMS), Mead‐irrigated continuous maize site (US‐Ne1), Mead‐irrigated maize‐soybean rotation site (US‐Ne2), Mead‐rainfed maize‐soybean rotation site (US‐Ne3), Niwot Ridge Forest (US‐NR1), Park Falls (US‐PFa), Santa Rita Grassland (US‐SRG), Sanata Rita Mesquite (US‐SRM), Tonzi Ranch (US‐Ton), University of Michigan Biological Station (US‐UMB), University of Michigan Biological Disturbance (US‐UMd), Vaira Ranch‐ Ione (US‐Var), Willow Creek (US‐WCr), Walnut Gulch Lucky Hills Shrub (US‐Whs), and Walnut Gulch Kendall Grasslands (US‐Wkg). These data were obtained online (from https://fluxnet.org).

## 3 Methods

We focus our analysis on quantifying the relative contribution of amplification and compensation to IAV in NEE and GPP over temperate North America. First, we define how anomalies are calculated (section 3.1), and then we introduce two metrics for quantifying amplification and compensation in IAV (section 3.2). We also show that taking the ratio of the magnitude of compensation to the magnitude of amplification provides a metric of the relative contribution of each quantity to IAV. Finally, we introduce how singular value decomposition (SVD) can be employed to extract the dominant modes of IAV between years (section 3.3), which can then be compared with the metrics of amplification and compensation.

### 3.1 Definition of Anomalies

Anomalies are denoted with a “Δ” for all quantities (e.g., ΔNEE). To calculate anomalies, the mean seasonal cycle over a baseline period is removed. The baseline period employed is 2010–2015 for flux inversion NEE, 2003–2014 for GRACE TWS, and 2001–2017 for GPP, soil temperature, soil moisture, and precipitation. In addition, a linear trend is removed for all data sets except the NEE flux inversion (because the flux inversion time series is only 6 years). Sensitivity tests found that results were not sensitive to the time period chosen for the baseline.

### 3.2 Quantifying IAV Features

We focus our analysis on the seasonal compensation component and amplification component of IAV over the growing season. For NEE, we define the seasonal compensation component (NEE_comp_) and seasonal amplification component (NEE_amp_) as

(1) $$\Delta {\text{NEE}}_{\text{comp}}=\Delta {\text{NEE}}_{\text{Jul‐Aug‐Sep}}-\Delta {\text{NEE}}_{\text{Apr‐May‐Jun}},$$

(2) $$\Delta {\text{NEE}}_{\text{amp}}=\Delta {\text{NEE}}_{\text{Jul‐Aug‐Sep}}+\Delta {\text{NEE}}_{\text{Apr‐May‐Jun}},$$

where ΔNEE_Apr‐May‐Jun_ and ΔNEE_Jul‐Aug‐Sep_ are the mean anomalies across April–June and July–September, respectively. A schematic of NEE anomalies leading to positive and negative amplification and compensation components are shown in Figure S1. The amplification component indicates a net increase or decrease in carbon uptake over the growing season. For example, if NEE anomalies are positive across the growing season (Figure S1a), this will imply positive amplification and enhanced CO_2_ emitted to the atmosphere (ΔNEE_amp_ > 0). The compensation component indicates anticorrelated anomalies between the spring and summer. For example, if NEE anomalies are positive in the spring but negative in the summer (Figure S1b), this will imply a negative compensation over the growing season (ΔNEE_comp_ < 0). We define compensation and amplification for GPP in the same way.

We examine the relative magnitudes of these two components by taking the ratio of the mean absolute seasonal compensation component to the mean absolute amplification component. For NEE, this ratio is defined as

(3) $${\text{NEE}}_{\text{RATIO}}=\frac{\overset{2015}{\underset{y=2010}{\sum }}|\Delta NEE_{comp}|}{\overset{2015}{\underset{y=2010}{\sum }}|\Delta NEE_{amp}|}.$$

The quantity NEE_RATIO_ provides a measure of the relative magnitudes of the compensation and amplification components. An NEE_RATIO_ of one indicates that the amplification and compensation components are of equal magnitude. If the magnitude of compensation is generally larger than amplification, then the ratio will be larger than one. If amplification dominates, then the ratio will be less than one. The motivation for examining these components as a ratio is that it removes the dependence of the absolute magnitudes of IAV. In this analysis, we are most interested in examining relative differences in this NEE_RATIO_ across temperate North America. That is, we aim to determine which regions have a larger component of seasonal compensation relative to the amplification component and what ecological and environmental variables drive spatial structures. It should be noted that this metric could result in very large values when the magnitude of amplification is very small. A similar metric developed by Butterfield et al. (2020) addresses this issue by examining the ratio of the mean anomaly across a number months relative to the mean of the absolute anomaly for each month. However, we feel that NEE_RATIO_ more directly compares the compensation and amplification components as defined in this study.

Note that we split the growing season into the spring (April–June) and summer (July–September). The spring roughly covers the period from the spring equinox (March 20) to the summer solstice (June 20), while the summer roughly covers the period from the summer solstice to the fall equinox (September 22). We note that these definitions are lagged by 1 month from the meteorological seasons.

### 3.3 Singular Value Decomposition

We employ SVD to examine the modes of variability in monthly ΔNEE and ΔGPP between years. SVD is a method to decompose a matrix into a set of singular vectors and singular values (Golub & Reinsch, 1971), where the singular vectors are a set of orthogonal basis vectors. In plain english, this is a method that performs a linear transformation to a coordinate system that most simply explains the data within a matrix, with the first singular vector explaining the largest fraction of variability within the matrix. In this analysis, we perform SVD on ΔGPP and ΔNEE arranged into month‐by‐year matrices. Thus, the singular vectors give the modes of monthly variability between years in ΔGPP and ΔNEE. The fraction of overall variance explained by the leading singular vector “*i*” is then calculated using the expression $R^{2}=s_{i}^{2}/\sum_{j}s_{j}^{2}$, where *s*_*j*_ are the singular values.

## 4 Results

### 4.1 Amplification Dominates in the West and Compensation Dominates in the East

We examine seasonal compensation and amplification in ΔGPP and ΔNEE over temperate North America in two steps. First, we look at the relative magnitudes of compensation and amplifications at high spatial resolution (4° × 5° grid cells). It is important to emphasize that we do not expect that the CO_2_ flux inversions fully recover NEE IAV at this spatial scale. Instead, we employ this analysis to examine the large‐scale spatial structures of amplification and compensation over temperate North America. Second, we aggregate the NEE and GPP anomalies into large spatial regions and perform SVD analysis to determine the dominant modes of IAV. We then compare the dominant modes of IAV in the data to the amplification and compensation metrics of IAV.

**Figure 1:**
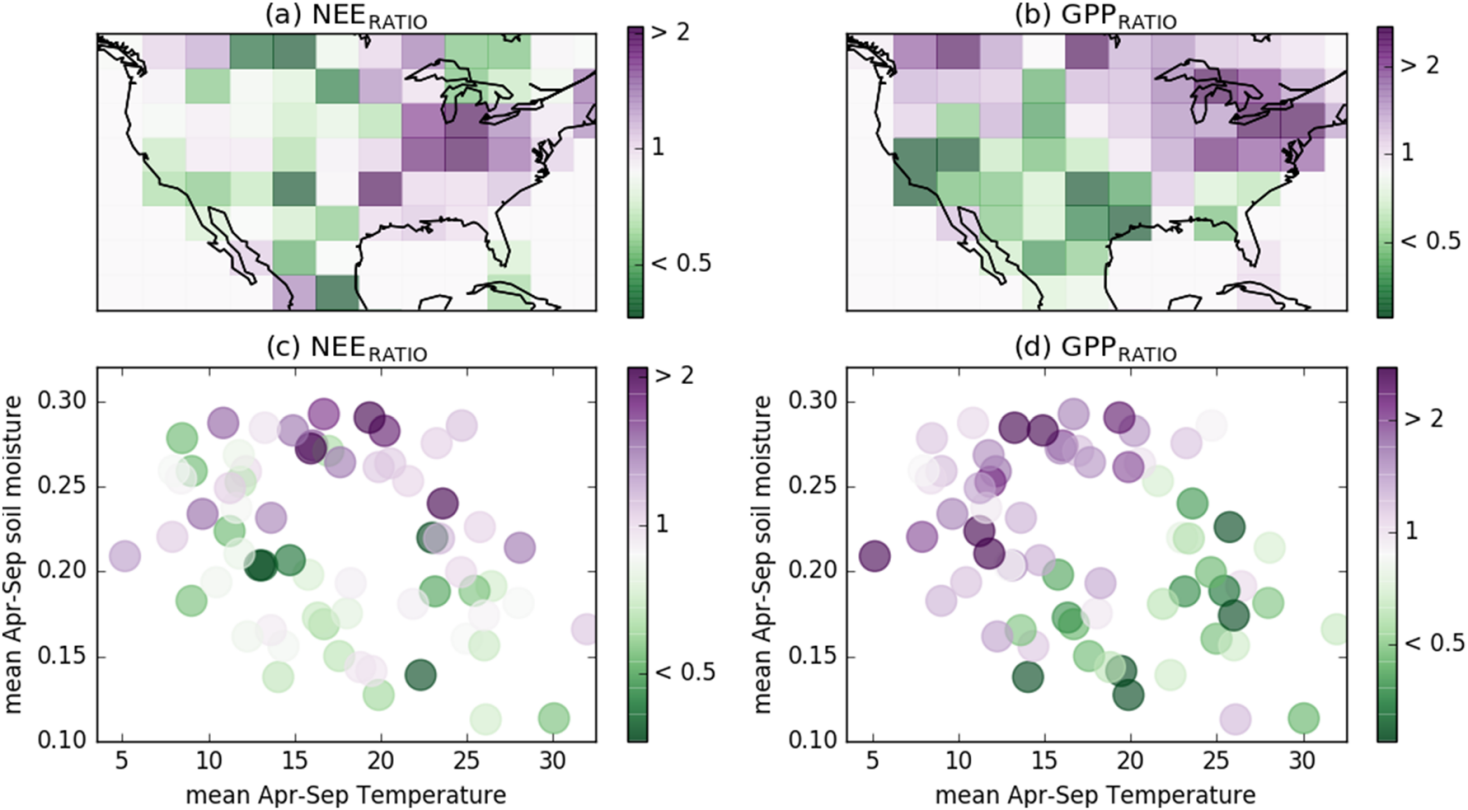
Relative magnitudes of seasonal compensation and amplification. (a) NEE_RATIO_ over 2010–2015 and (b) GPP_RATIO_ over 2001–2017 at 4° × 5°. (c) NEE_RATIO_ and (d) GPP_RATIO_ plotted as a function of April–September mean soil temperature (K) and soil moisture (m^3^ m^−3^).

Figure 1 shows NEE_RATIO_ for 2010–2015 and GPP_RATIO_ for 2001–2017 over subtropical and temperate North America at 4° × 5° spatial resolution (GPP_RATIO_ for 2010–2015 is shown in Figure S2). A ratio of one indicates that the magnitude of the compensation and amplification components are equal. Larger ratios indicate that the magnitude of the compensation component is larger, while ratios less than one imply the opposite. Spatially, seasonal compensation is most dominant in eastern temperate North America (largest ratios), particularly around the Midwest. In contrast, the amplification component of IAV is most dominant in western temperate North America, particularly in the southwest. Figures 1c and 1d show NEE_RATIO_ and GPP_RATIO_ as a function of the mean April–September soil moisture and soil temperature for each 4° × 5° grid cell. Larger ratios are found to cluster in the wetter areas while smaller ratios are generally found in the drier areas, consistent with the climatological difference between the west and east of temperate North America.

**Figure 2:**
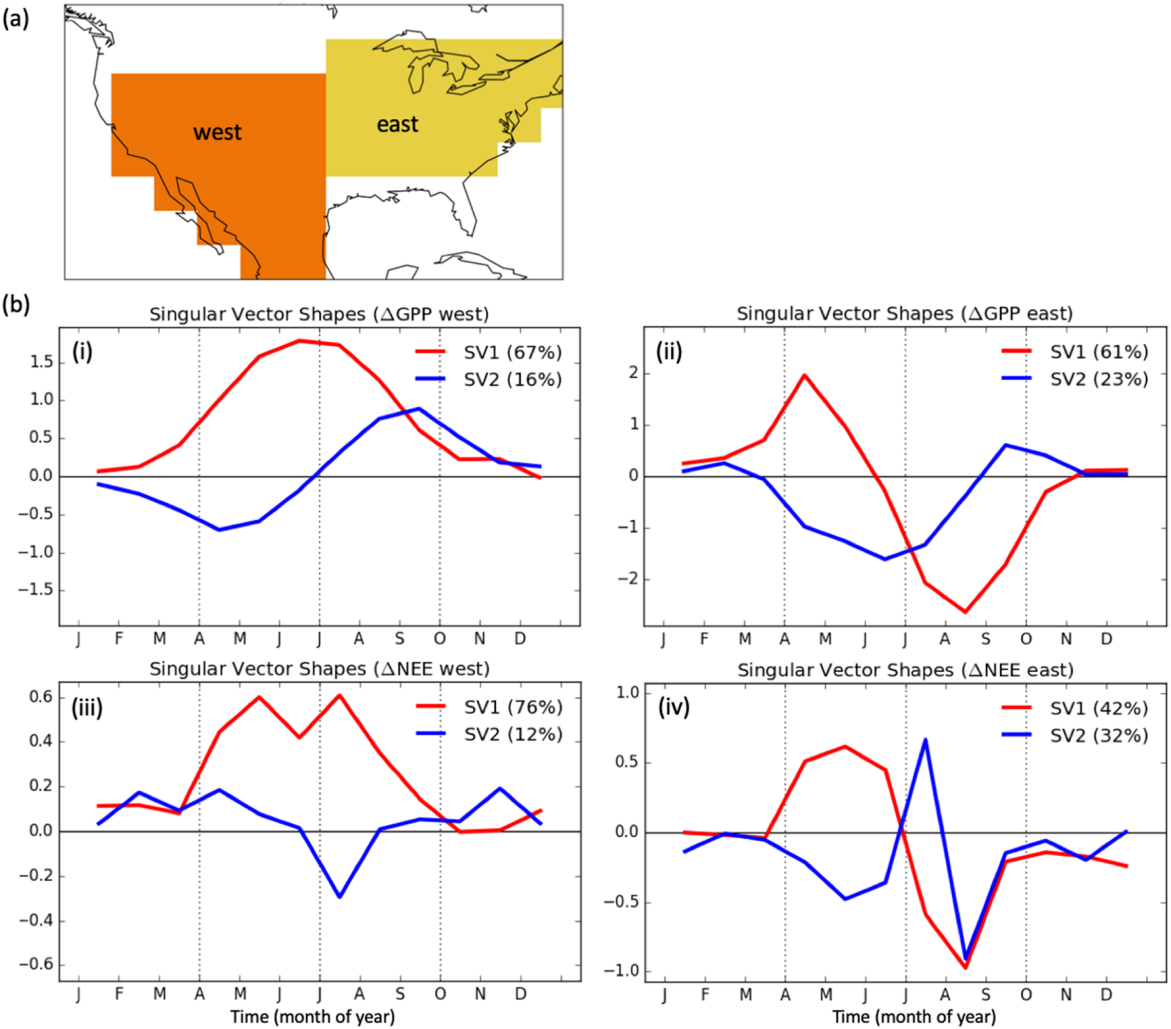
(a) The spatial extent of western (orange) and eastern (yellow) regions of temperate North America. (b) First and second singular vectors resulting from the decomposition of the IAV in GPP over 2001–2017 for the (i) western and (ii) eastern regions of temperate North America and of the IAV in NEE over 2010–2015 for the (iii) western and (iv) eastern regions of temperate North America.

To further examine differences in IAV between eastern and western temperate North America, we aggregate grid cells into western and eastern regions (Figure 2a). We then perform SVD on matrices of monthly ΔNEE and ΔGPP (with months as the rows and years as columns) over these two regions. This analysis allows us to compute basis vectors that explain modes of variability in monthly ΔNEE and ΔGPP between years. The first and second basis vectors, which explain the majority of variability in ΔNEE and ΔGPP are shown in Figure 2. In the west, the first basis vector shows amplification structure (with correlated anomalies between spring and summer) for both GPP and NEE. Furthermore, this first basis explains the majority of variability in NEE and GPP between years, as the first singular value explains 66% and 76% of the variance for GPP and NEE, respectively (Figure 2). Conversely, the eastern region is dominated by seasonal compensation in GPP and NEE. The first singular vector has a compensation shape, where positive anomalies in the spring are associated with negative anomalies in the summer. This mode of variability explains the majority of year‐to‐year variability for GPP (59%) and about half of the variability for NEE (47%) (Figure 2). Thus, these aggregated regions are generally reflective of the IAV seen at the grid cell level, showing amplification is dominant in the west and compensation is dominant in the east. We further examine the robustness of the NEE SVD analysis by performing the SVD analysis on each of the three individual inversions from Byrne et al. (2020) (Figure S3). We find consistent results, where the first singular vector is amplification‐like in the west (explaining 59–83% of the variance) and compensation‐like in the east (explaining 37–47% of the variance).

### 4.2 East‐West NEE Differences Seen in Multiple Data Sets

The NEE fluxes employed in this study only cover a 6‐year period; thus, is it possible that the results found here are specific to this period and are not generalizable across time. In this section, we compare the relative magnitudes of amplifications and compensation in NEE for several flux inversions and for FLUXNET eddy covariance sites, which cover a variety of time periods.

The NEE fluxes used in this analysis are unique, in that they incorporate CO_2_ observational constraints of space‐based $X_{{\text{CO}}_{2}}$ from the Greenhouse Gases Observing Satellite (GOSAT), surface‐based $X_{{\text{CO}}_{2}}$ measurements from the total column carbon observing network (TCCON), and CO_2_ measurements from the network of flask and in situ sites. This type of inversion is temporally limited due the fact that GOSAT was launched in 2009. Byrne et al. (2020) argue that this combined flux inversion (referred to as “GOSAT + surface + TCCON”) provides improved CO_2_ flux estimates relative to flux inversions that only assimilate flask and in situ measurements (referred to as “surface only”). Therefore, we may expect that flask and in situ CO_2_ flux inversions may not separate IAV between eastern and western temperate North America as distinctly. Nevertheless, we examine whether similar east‐west differences are seen for a series of in situ and flask flux inversions.

**Figure 3:**
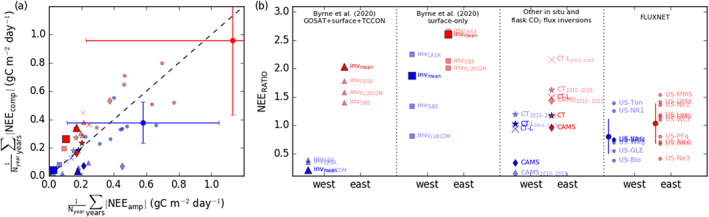
(a) Mean magnitude of NEE compensation versus mean magnitude of NEE amplification across multiple years. (b) NEE_RATIO_ over eastern and western temperate North America for (left‐to‐right) the combined GOSAT+surface+TCCON flux inversions of Byrne et al. (2020), the surface‐only flux inversions of Byrne et al. (2020), three independent flux inversions (CT2017, CT‐L, and CAMS) that assimilate flask and in situ CO_2_ measurements, and FLUXNET sites with 6+ years of data within the eastern and western domains. Partially transparent symbols show values over 2010–2015 and solid colors are for the entire time period examined in this study for a given dataset.

Figure 3 shows the mean magnitude of the amplification components, compensation components, and NEE_RATIO_ for a set of flux inversions and FLUXNET sites. The set of GOSAT + surface + TCCON fluxes inversions from Byrne et al. (2020) (three inversion setups and ensemble mean) show distinct differences between eastern and western temperate North America. The surface‐only flux inversions also show differences between eastern and western temperate North America, but differences are reduced, and scatter between inversions is increased, suggesting that the lower data density of assimilated observation reduces the ability of the inversion to isolate east‐west differences.

Next, we examine a set of independent flask and in situ flux inversions that extend over larger time spans: CarbonTracker version CT2017 covering 2000–2016, CT‐L covering 2007–2015 (Hu et al., 2019), and CAMS covering 2000–2018. For each flux inversion, we examine the posterior fluxes over 2010–2015 and over the entire period. We find that all inversions show greater NEE_RATIO_ in the east than the west. However, we also find that the 2010–2015 period generally shows larger east‐west differences. In particular, the NEE_RATIO_ is increased in the east during 2010–2015, likely due to the temperate North American drought of 2012 (Liu, Bowman, Parazoo, et al., 2018; Wolf et al., 2016).

Finally, we examine east‐west differences for FLUXNET sites within the two regions, including sites with six or more full years of data. In the western domain, we include US‐Blo, US‐GLE, US‐NR1, US‐SRG, US‐SRM, US‐Ton, US‐Var, US‐Whs, and US‐Wkg. In the eastern domain, we include US‐ARM, US‐Los, US‐MMS, US‐Ne1, US‐Ne2, US‐Ne3, US‐UMd, US‐UMB, and US‐WCr. There is considerable scatter between FLUXNET sites for each of the metrics examined. However, taking the mean and standard deviation of NEE_RATIO_ for sites in east and west, we find larger values in the east relative to the west, consistent with the flux inversion.

Across the set of NEE estimates examined here, we consistently find that the compensation component of IAV is greater relative to the amplification component in eastern temperate North America. Therefore, we find the results found for the GOSAT + surface + TCCON NEE fluxes examined in this study are generally supported by independent flux estimates across different time periods.

Similar analysis is performed for FluxSat GPP, GOME‐2 SIF, MODIS NDVI, FLUXCOM GPP, and FLUXNET GPP in the supporting information (Figure S4). We find the remote sensing products show similar east‐west differences, with larger GPP_RATIO_ in the east. However, both FLUXCOM and FLUXNET GPP do not show substantial east‐west differences. In general, FLUXNET sites do not show a coherent response within each region, which is probably at least partially due to the fact that they are site level observations rather than a large scale average. In a comparison of IAV in ecosystem productivity by remote sensing and eddy covariance, Butterfield et al. (2020) found that FLUXNET sites generally showed less coherent patterns in IAV than the large‐scale averaged patterns obtained from remote sensing products. FLUXCOM GPP exhibits very weak IAV across the regions examined here, which may partially explain why it does not show clear east‐west differences.

### 4.3 Relationship Between Flux Anomalies and Environmental Drivers

**Figure 4:**
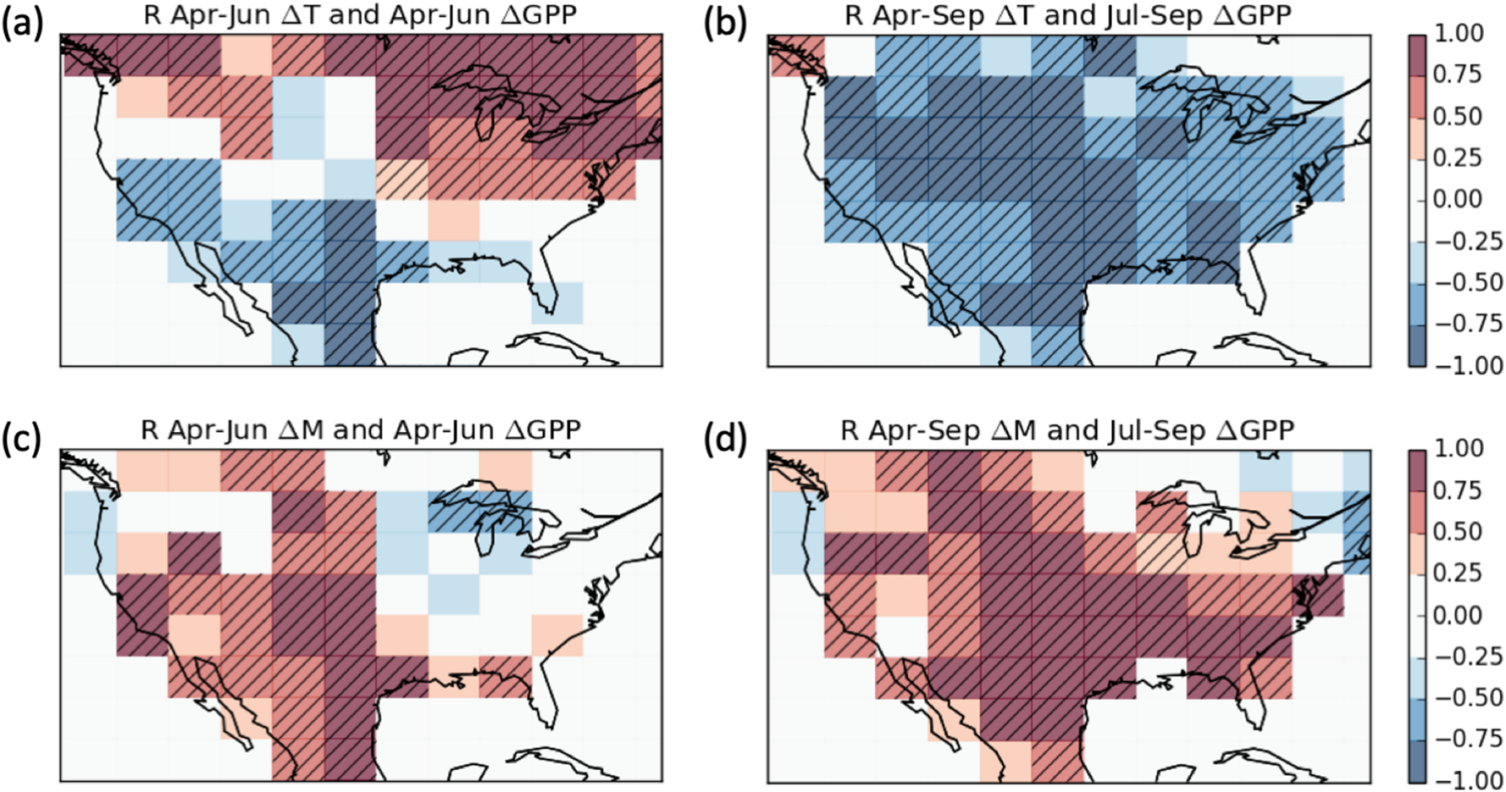
Relationship between ΔGPP and variations in climate. Coefficient of correlation (*R*) over 2001–2017 for 4° × 5° grid cells between (a) April–June ΔT and April–June ΔGPP, (b) April–September ΔT and July–September ΔGPP, (c) April–June ΔM and April–June ΔGPP, and (d) April–September ΔM and July–September ΔGPP. Hatching shows grid cells for which *P* < 0.05.

To a large extent, IAV in the carbon balance of ecosystems is expected to be driven by IAV in temperature and moisture (Berry & Bjorkman, 1980; Byrne et al., 2019; Smith et al., 2011); thus, we examine the relationship between CO_2_ flux anomalies and anomalies in soil temperature (ΔT) and soil moisture (ΔM). Figure 4 shows the correlation between ΔGPP and anomalies in climate variables over 2001–2017. Note that we correlated July–September flux anomalies with April–September climate anomalies to incorporate lagged effects of spring climate anomalies on summer carbon cycle anomalies. We find spatial differences in the correlation coefficient between western and eastern temperate North America. In the west, increased GPP (positive ΔGPP) is found to be correlated with cooler (negative ΔT) and wetter (positive ΔM) conditions during both April–June and July–September. The temporally coherent relationship between flux anomalies and environmental anomalies in western temperate North America suggests that cooler‐wetter years will lead to an amplification of carbon uptake. In the east, increased GPP is correlated with warmer conditions during April–June, but cooler and wetter conditions during July–September. These seasonal variations in the relationship between flux anomalies and environmental variables suggest that seasonal compensation will occur when climate anomalies persist throughout the year. For example, warm years would result in increased uptake during the spring but decreased uptake during the summer. Similar results are found for NEE (Figure S5) over 2010–2015, although correlations are generally less statistically significant. This is likely partially explained by the shorter time period examined and the inability of the flux inversion to isolate NEE anomalies to 4° × 5° spatial grid cells.

**Figure 5:**
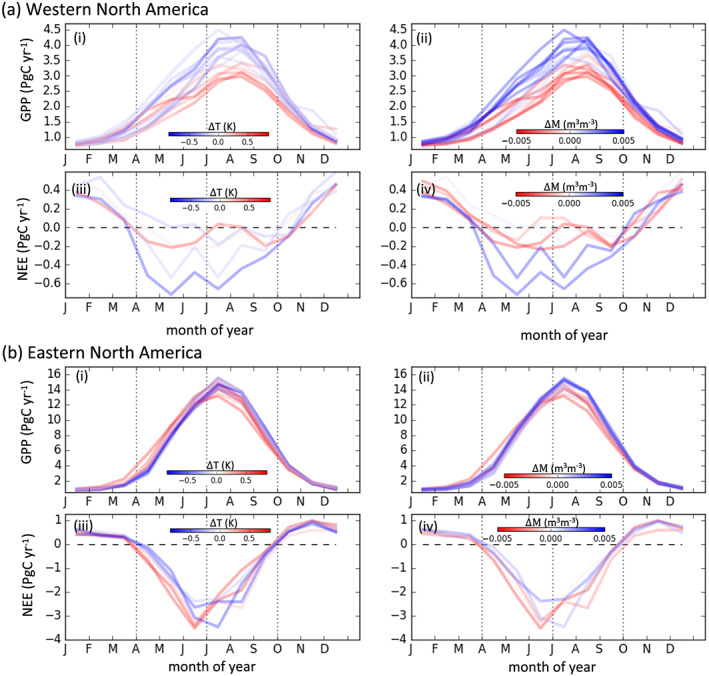
Seasonal cycles of GPP (2001–2017) and NEE (2010–2015) over eastern and western temperate North America. (a) Seasonal cycles of (i and ii) GPP and (iii and iv) NEE over western temperate North America. (b) Seasonal cycles of (i and ii) GPP and (iii and iv) NEE over eastern temperate North America. Colors indicate the April–September ΔT (i and iii) or April–September ΔM (ii and iv).

We now examine the seasonal cycles of GPP and NEE over the western and eastern regions of temperate North America. Figure 5 shows the seasonal cycles of GPP (2001–2017) and NEE (2010–2015) over the western and eastern regions of temperate North America with different years colored by the corresponding April‐September ΔT or ΔM. An additional plot showing the seasonal compensation and amplification components as a function of ΔT or ΔM is shown in the supporting information (Figure S6). For western temperate North America, variations in the seasonal cycle of GPP and NEE are dominated by an amplification component over April–September. Increased GPP and net uptake are associated with cooler and wetter conditions. ΔT and ΔM are strongly correlated with each other ( R=−0.77 for 2001–2017), obscuring which variable has the largest impact on IAV. However, the magnitude of the correlation is slightly larger for ΔM as compared with ΔT for ΔNEE_amp_ (0.91 vs. 0.71) and ΔGPP_amp_ (0.66 vs. 0.63) (Table S1). IAV is generally weaker in eastern temperate North America (relative to the mean seasonal cycle). Temporal shifts in the seasonal cycle of GPP (ΔGPP_comp_) and NEE (ΔNEE_comp_) provide the largest component of IAV. Shifts of GPP and NEE to earlier in the year are associated with positive Apr‐Sep ΔT (Figure 5b, i and iii), suggesting that a warm spring drives the shift and persistent warming during summer reduces the productivity and net uptake. Variations in April–September ΔM are more closely tied to an amplification component of ΔGPP ( R=0.72) and ΔNEE ( R=0.78) (Table S1). This implies that increased soil moisture is associated with increased GPP but reduced net uptake, suggesting that respiration fluxes increase more than GPP with increased soil moisture. This result is consistent with Liu, Ballantyne, Poulter, et al. (2018) but contradicted (for droughts) by Schwalm et al. (2010). Thus, more research is needed on this topic.

### 4.4 Impact of Amplification and Compensation for Net CO_2_ Fluxes

**Figure 6:**
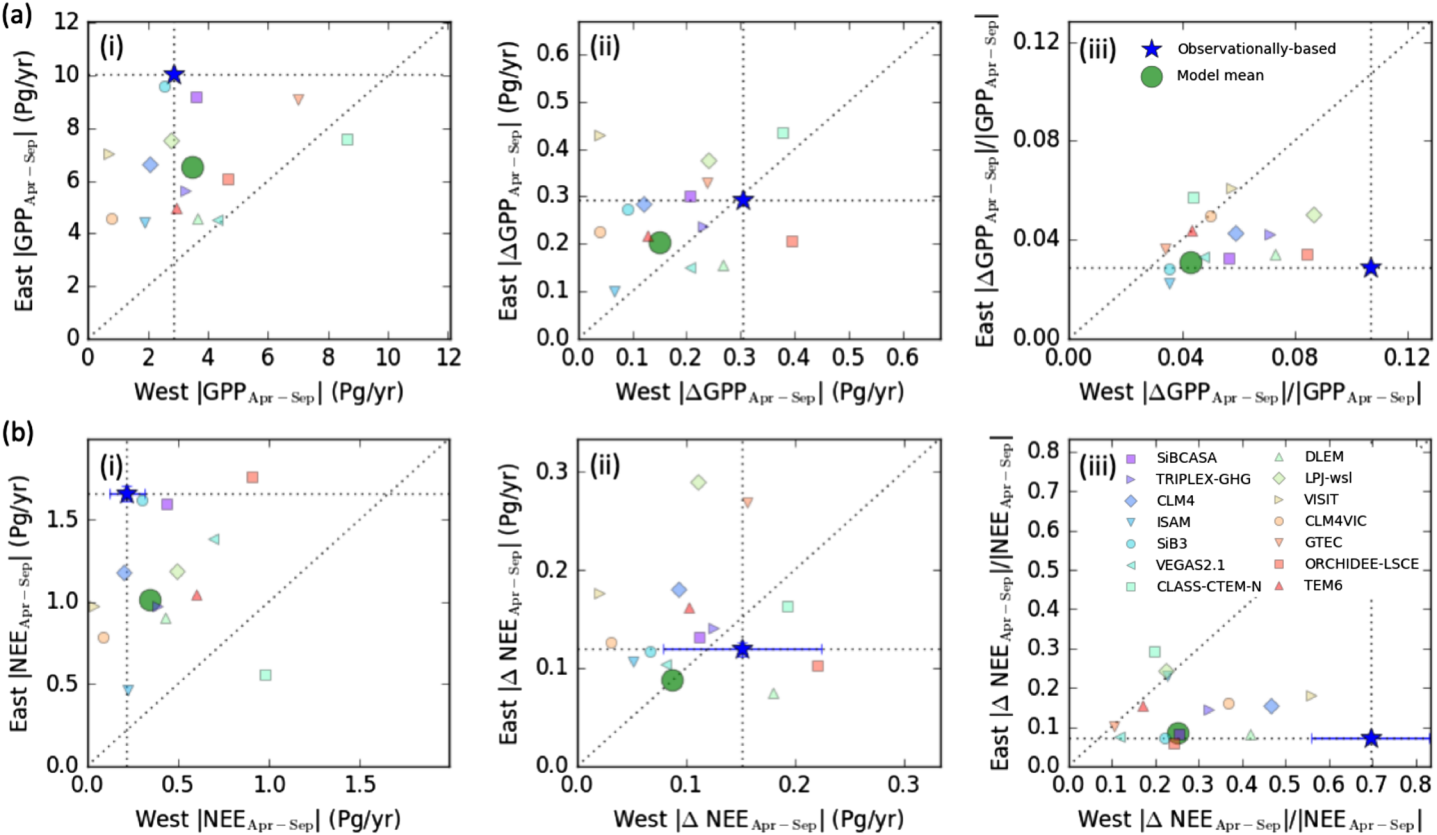
Scatter plots of (a) GPP and (b) NEE fluxes in eastern and western temperate North America. The panels show (i) the magnitude of April–September mean fluxes, (ii) the magnitude of April–September mean anomalies, and (iii) the ratio of the anomalies to mean fluxes. The blue star shows the observationally based estimates from FluxSat GPP and the flux inversion NEE. The error bars on the observationally constrained NEE estimate show the range in these values between the three flux inversions from Byrne et al. (2020); note that error bars are very small for the east. The large green circle shows the GPP and NEE estimate from the MsTMIP model mean. Small symbols show the GPP and NEE estimates from individual MsTMIP models.

The presence of temporally coherent spring‐summer flux anomalies in western temperate North America acts to increase the annual net flux anomalies. In contrast, anticorrelated spring‐summer flux anomalies in eastern temperate North America act to reduce the net annual flux anomalies. Here we examine the relative contribution of eastern and western temperate North America to the mean seasonal cycle and anomalies of GPP and NEE (Figure 6). We find that monthly NEE and GPP fluxes are larger in eastern temperate North America than in western temperate North America (7.6× larger in east than west for GPP, 3.5× for NEE), reflecting a more productive carbon cycle. However, due to seasonal compensating anomalies, annual anomalies in GPP and NEE are larger in the west than the east (1.04× larger in west than east for GPP and 1.27× for NEE). Thus, growing season IAV in NEE and GPP is larger in the western temperate North America, despite a more productive carbon cycle in eastern temperate North America. The impacts of these differences in IAV between these two regions are evident in the time series of ΔGPP and ΔNEE anomalies for the two regions (Figure S7). Monthly anomalies in western temperate North America are coherent for individual years leading to increased annual anomalies, while anomalies in the east show seasonal compensation, reducing annual net anomalies.

We now investigate the ability of the MsTMIP models to recover observationally constrained east‐west differences in GPP and NEE over 1980–2010. Modeled fluxes are plotted with the observationally constrained estimates in Figure 6. The MsTMIP models systematically underestimate the magnitude of April–September GPP and NEE in eastern temperate North America relative to FluxSat GPP and inversion NEE but closely agree with the observationally constrained fluxes in western temperate North America. The mean magnitudes of April–September ΔGPP and ΔNEE are variable between MsTMIP models but are generally smaller than the observationally based estimates. The model mean gives similar magnitudes of ΔGPP and ΔNEE in eastern and western temperate North America, suggesting that the models at least partially capture increased IAV in western temperate North America. The ratio of the magnitudes of April–September IAV to the April–September mean are shown in Figure 6, iii. The models systematically underestimate this ratio for GPP and NEE in western temperate North America. The MsTMIP models predict that mean magnitude of April–September ΔGPP is 4% (range of 3–9%) of the April–September GPP, while FluxSat GPP suggests 11%. Similarly, MsTMIP models predict that mean magnitude of April–September ΔNEE is 25% (range of 11–56%) of the April–September NEE, while inversion NEE suggests 70%. The MsTMIP model mean GPP gives weaker sensitivity to soil moisture and temperature anomalies than FluxSat GPP, which is found to be about 30% more sensitive (Table 2). Inversion NEE sensitivities are consistent with the MsTMIP model mean NEE but are also quite uncertain (indicated by the range in sensitivities between individual flux inversions using SiB3, CASA, or FLUXCOM as priors). In eastern temperate North America, the MsTMIP models suggest greater sensitivity to environmental variables than the observationally constrained fluxes (Table 2), as previously suggested by Shiga et al. (2018).

**Table 2:** Observationally Based and Model‐Based Sensitivities

	West				East			
	Temperature		Soil moisture		Temperature		Soil moisture	
	slope (PgC K^−1^)	*R*^2^	slope (PgC (m^3^ m^−3^)^−1^)	*R*^2^	slope PgC K^−1^	*R*^2^	slope (PgC (m^3^ m^−3^)^−1^)	*R*^2^
FluxSat ΔGPP	−0.29	0.44	**32.6**	**0.89**	−0.04	0.03	52.2	0.09
Model ΔGPP	− **0.20**	**0.55**	**23.4**	**0.91**	−0.02	0.02	**110.6**	**0.45**
Inversion ΔNEE	0.13	0.47	−10.3	0.49	−0.04	0.19	28.6	0.21
(range)	(0.06–0.19)	(0.36–0.53)	(−14.6 to −4.6)	(0.37–0.71)	(−0.03 to 0.06)	(0.15–0.60)	(−53.47 to 28.0)	(0.10–0.42)
Model ΔNEE	**0.11**	**0.53**	− **10.3**	**0.71**	**0.06**	**0.60**	− **53.5**	**0.42**

*Note*. Slope and *R*^2^ values for linear regressions of April–September ΔGPP and ΔNEE against April–September ΔT and ΔM for FluxSat GPP (2001–2017), inversion NEE (2010–2016), and MsTMIP model mean GPP and NEE (2001–2010). A range is provided for the inversion ΔNEE indicating the range for each individual inversion with different prior fluxes. MsTMIP fluxes are examined over 2001–2010 to isolate comparisons to the period when observational data sets are best constrained by observations. Bold numbers indicate *P* < 0.05.

It should be noted that IAV for the MsTMIP ensemble, FluxSat GPP and flux inversion NEE are calculated over different baselines. As shown in section 4.2, the magnitude of amplification and compensation does show some sensitivity to the baseline years from which the anomalies are calculated. Therefore, it is possible that some of the difference seen between observationally constrained estimates and the MsTMIP ensemble are due to differences in the baseline. Unfortunately, the time periods of these data sets do not overlap, and we are limited to a 6‐year period for the NEE estimates from Byrne et al. (2020). Ongoing research is working toward building decadal‐scale records of NEE from space‐based CO_2_ observations (Liu, Baskarran, et al., 2020). Thus, we expect that future studies that will be able to more precisely identify differences in IAV between TBMs and observationally constrained estimates over the same time period.

## 5 Discussion

### 5.1 Mechanisms Driving IAV

#### 5.1.1 Western Temperate North America

We find that IAV in western temperate North America is dominated by an amplification component, wherein increased GPP and net uptake are associated with cooler‐wetter conditions through the entire growing season. This result is consistent with a number of previous studies investigating southwest temperate North America (Hu et al., 2019; Papagiannopoulou et al., 2017; Parazoo et al., 2015; Shiga et al., 2018; Zhang et al., 2013). Variations in GPP and NEE over this region are likely primarily due to variations in water availability, rather than temperature variability (Papagiannopoulou et al., 2017). Parazoo et al. (2015) have shown that variability in productivity over the Southern U.S.‐Northern Mexico region is linked to El Niño‐Southern Oscillation (ENSO) and the North Atlantic Oscillation (NAO) and suggest that year‐to‐year variability of carbon net uptake is associated with precipitation anomalies in this region. We find ΔP is strongly correlated with ΔGPP_amp_ ( R=0.78) and moderately correlated with ΔNEE_amp_ ( R=−0.47) in western temperate North America (Table S1). This suggests that IAV in western temperate North America is primarily driven by large‐scale climate variability. Supporting this result, Hu et al. (2019) found that temperate North American net uptake is correlated with ENSO phase, which they primarily attributed to variations in water availability.

#### 5.1.2 Eastern Temperate North America

We find that GPP and NEE IAV in eastern temperate North America is dominated by a seasonal compensation component, where an increase in April–June is followed by a compensating decrease in July–September. This is most closely linked to a shift of the seasonal cycle to earlier in the year with increased temperature. This phenomenon has previously been reported for studies of phenology (Fu et al., 2014; Keenan & Richardson, 2015), GPP (Butterfield et al., 2020; Buermann et al., 2013, 2018; Papagiannopoulou et al., 2017; Parida & Buermann, 2014), and NEE (Hu et al., 2019; Liu, Bowman, Parazoo, et al., 2018; Rödenbeck et al., 2018; Shiga et al., 2018; Wolf et al., 2016). Most studies attribute this phenomena to land‐atmosphere interactions, wherein a warm spring results in drying and drought during the summer (Parida & Buermann, 2014; Wolf et al., 2016). This explanation is generally consistent with our results for GPP but not for NEE. We find that April–June ΔGPP and ΔNEE are correlated with April–June ΔT ( R=0.86 for GPP and R=−0.95 for NEE) but only July–September ΔGPP is correlated with July–September ΔM ( R=0.72 for GPP and R=0.16 for NEE). Furthermore, this mechanism would imply a negative correlation between spring ΔT and summer ΔM; however, April–June ΔT and July–September ΔM are only weakly correlated over eastern temperate North America ( R=−0.28). This is true for grid cells with cropland fractions greater than 65% ( R=−0.19) and less than 35% ( R=−0.28) (see Figure S8). To some extent, the lack of correlation could be due to errors in the ESA CCI soil moisture product, as somewhat stronger correlations are found between April–June ΔT and July–September GRACE ΔTWS ( R=−0.44 for 2003–2014; Table S1). Still, these results suggests that other factors play a role in seasonal compensation effects. Direct physiological mechanisms linking budburst and senescence, such as leaf structure constraints on longevity (Reich et al., 1992) or programmed cell death (Lam, 2004), may have a significant impact on the length of the growing season (Keenan & Richardson, 2015). However, more research is needed to understand the drivers of seasonal compensation effects.

### 5.2 Implications for Temperate North American Carbon Sink

The sensitivity of carbon cycle IAV to environmental drivers may provide information on the sensitivity of the carbon cycle to climate change (Cox et al., 2013). Here, we discuss the implications of the relationships between carbon cycle IAV and environmental drivers for the future carbon balance of temperate North America under anthropogenic climate change.

Changes in temperature and the water cycle of temperate North America have been observed and are projected into the future. The annual average temperature of the contiguous United States has risen by 0.7–1.0°C since the start of the 20th century and is projected to increase by 1.4°C (RCP4.5) to 1.6°C (RCP8.5) for 2021–2050 relative to 1976–2005, based on Coupled Model Intercomparison Project 5 (CMIP5) simulations (Vose et al., 2017). Warming is driving a more rapid water cycle (Huntington et al., 2018). This is projected to cause decreases in soil moisture because increases in evapotranspiration (due to temperature increases) are expected to be larger than precipitation increases (Cook et al., 2015). Predicted warming and drying in western temperate North America (Seager et al., 2007) could have profound effects on the carbon cycle (Schwalm et al., 2012), with increasing temperatures and aridity driving reductions in growing season productivity and carbon uptake, although TBMs suggest that carbon loss due to climate change will be partially mitigated by increasing CO_2_ (Huntzinger, Chatterjee, et al., 2018). In eastern temperate North America, the results of this study suggest that temperature increases will result in a shift of the growing season to earlier in the year, with increased uptake during the spring but decreased uptake during the summer. However, the observationally constrained flux estimates do not show sensitivity of growing season net GPP and NEE to environmental anomalies, suggesting that eastern temperate North American ecosystems may be more resilient to climate change than simulated by the models.

## 6 Conclusions

Observationally‐constrained FluxSat GPP and CO_2_ flux inversion NEE show that there are substantial differences in IAV between the arid west and wetter east of temperate North America. In western temperate North America, spring and summer anomalies are found to be correlated, such that IAV is characterized by an amplification of the mean GPP and NEE during the growing season. These western ecosystems are generally water limited, such that increased GPP and net uptake are associated with cooler‐wetter conditions. In eastern temperate North America, spring and summer anomalies are anti‐correlated, leading to compensating anomalies over the growing season. Anomalies in GPP and NEE are closely associated to temperature, with a shift in the seasonal cycle to earlier in the year during warm years, resulting in increased GPP and net uptake in Apr–Jun but decreased GPP and net uptake in Jun‐Sep.

Due to the dominance of amplification in the west and seasonal compensation in the east, western temperate North America contributes more to IAV than the eastern temperate North America in GPP (104% of east) and NEE (127% of east) during the growing season (April‐September), despite the fact that the mean growing season fluxes are larger in the east (7.6× for GPP, 3.5× for NEE). Simulated GPP and NEE from the MsTMIP ensemble generally recover larger IAV in the west relative to the east, although there is considerable spread between models. These results suggest that ecosystems in western temperate North America are sensitive to increases in temperature and aridity expected under climate change, and that reductions in growing season productivity and net uptake could occur under climate change.

## Supporting information



Supporting Information S1

## Data Availability

Posterior NEE fluxes from Byrne et al. (2020) were downloaded online (from https://cmsflux.jpl.nasa.gov/). CarbonTracker CT2017 results are provided by NOAA ESRL, Boulder, Colorado, USA (from the website http://carbontracker.noaa.gov). CarbonTracker Lagrange NEE fluxes were downloaded online (from https://doi.org/10.15138/3dw1‐5c37). CAMS NEE fluxes were obtained online (from https://atmosphere.copernicus.eu/). FLUXNET2015 data were obtained from the FLUXNET website (https://fluxnet.org).

## References

[gbc21045-bib-0001] Adler, R. F. , Huffman, G. J. , Chang, A. , Ferraro, R. , Xie, P.‐P. , Janowiak, J. , Rudolf, B. , Schneider, U. , Curtis, S. , Bolvin, D. , Gruber, A. , Susskind, J. , Arkin, P. , & Nelkin, E. (2003). The Version‐2 Global Precipitation Climatology Project (GPCP) monthly precipitation analysis (1979–present). Journal of Hydrometeorology, 4(6), 1147–1167.

[gbc21045-bib-0002] Ahlström, A. , Raupach, M. R. , Schurgers, G. , Smith, B. , Arneth, A. , Jung, M. , Reichstein, M. , Canadell, J. G. , Friedlingstein, P. , Jain, A. K. , Kato, E. , Poulter, B. , Sitch, S. , Stocker, B. D. , Viovy, N. , Wang, Y. P. , Wiltshire, A. , Zaehle, S. , & Zeng, N. (2015). The dominant role of semi‐arid ecosystems in the trend and variability of the land CO_2_ sink. Science, 348(6237), 895–899. 25999504 10.1126/science.aaa1668

[gbc21045-bib-0003] Angert, A. , Biraud, S. , Bonfils, C. , Henning, C. C. , Buermann, W. , Pinzon, J. , Tucker, C. J. , & Fung, I. (2005). Drier summers cancel out the CO_2_ uptake enhancement induced by warmer springs. Proceedings of the National Academy of Sciences, 102(31), 10,823–10,827. 10.1073/pnas.0501647102 PMC1180508 16043702

[gbc21045-bib-0004] Baldocchi, D. , Chu, H. , & Reichstein, M. (2018). Inter‐annual variability of net and gross ecosystem carbon fluxes: A review. Agricultural and Forest Meteorology, 249(Supplement C), 520–533. 10.1016/j.agrformet.2017.05.015

[gbc21045-bib-0005] Baldocchi, D. , Falge, E. , Gu, L. , Olson, R. , Hollinger, D. , Running, S. , Anthoni, P. , Bernhofer, C. , Davis, K. , Evans, R. , Fuentes, J. , Goldstein, A. , Katul, G. , Law, B. , Lee, X. , Malhi, Y. , Meyers, T. , Munger, W. , Oechel, W. , Paw, K. T. , Pilegaard, K. , Schmid, H. P. , Valentini, R. , Verma, S. , Vesala, T. , Wilson, K. , & Wofsy, S. (2001). FLUXNET: A new tool to study the temporal and spatial variability of ecosystem‐scale carbon dioxide, water vapor, and energy flux densities. Bulletin of the American Meteorological Society, 82(11), 2415–2434.

[gbc21045-bib-0006] Baldocchi, D. , & Ma, S. (2016). (2001‐2014) FLUXNET2015 US‐Ton Tonzi Ranch, Dataset. 10.18140/FLX/1440092

[gbc21045-bib-0007] Baldocchi, D. , Ma, S. , & Xu, L. (2016). (2000‐2014) FLUXNET2015 US‐Var Vaira Ranch‐ Ione, Dataset. 10.18140/FLX/1440094

[gbc21045-bib-0008] Baldocchi, D. , Ryu, Y. , & Keenan, T. (2016). Terrestrial carbon cycle variability. F1000Research, 5, 2371. 10.12688/f1000research.8962.1 PMC5040156 27746899

[gbc21045-bib-0009] Berry, J. , & Bjorkman, O. (1980). Photosynthetic response and adaptation to temperature in higher plants. Annual Review of Plant Physiology, 31(1), 491–543.

[gbc21045-bib-0010] Biederman, J. A. , Scott, R. L. , Arnone III, J. A. , Jasoni, R. L. , Litvak, M. E. , Moreo, M. T. , Papuga, S. A. , Ponce‐Campos, G. E. , Schreiner‐McGraw, A. P. , & Vivoni, E. R. (2018). Shrubland carbon sink depends upon winter water availability in the warm deserts of North America. Agricultural and Forest Meteorology, 249, 407–419.

[gbc21045-bib-0011] Biederman, J. A. , Scott, R. L. , Goulden, M. L. , Vargas, R. , Litvak, M. E. , Kolb, T. E. , Yepez, E. A. , Oechel, W. C. , Blanken, P. D. , Bell, T. W. , Garatuza Payan, J. , Maurer, G. E. , Dore, S. , & Burns, S. P. (2016). Terrestrial carbon balance in a drier world: The effects of water availability in southwestern North America. Global Change Biology, 22(5), 1867–1879. 26780862 10.1111/gcb.13222

[gbc21045-bib-0012] Biraud, S. , Fischer, M. , Chan, S. , & Torn, M. (2016). (2003‐2012) FLUXNET2015 US‐ARM ARM Southern Great Plains site‐ Lamont, Dataset. 10.18140/FLX/1440066

[gbc21045-bib-0013] Blanken, P. D. , Monson, R. K. , Burns, S. P. , Bowling, D. R. , & Turnipseed, A. A. (2016). (1998‐2014) FLUXNET2015 US‐NR1 Niwot Ridge Forest (LTER NWT1), Dataset. 10.18140/FLX/1440087

[gbc21045-bib-0014] Bowman, K. W. , Liu, J. , Bloom, A. A. , Parazoo, N. C. , Lee, M. , Jiang, Z. , Menemenlis, D. , Gierach, M. M. , Collatz, G. J. , Gurney, K. R. , & Wunch, D. (2017). Global and Brazilian carbon response to El Niño Modoki 2011–2010. Earth and Space Science, 4(10), 637–660. 10.1002/2016EA000204

[gbc21045-bib-0015] Buermann, W. , Bikash, P. R. , Jung, M. , Burn, D. H. , & Reichstein, M. (2013). Earlier springs decrease peak summer productivity in North American boreal forests. Environmental Research Letters, 8(2), 024027.

[gbc21045-bib-0016] Buermann, W. , Forkel, M. , O'Sullivan, M. , Sitch, S. , Friedlingstein, P. , Haverd, V. , Jain, A. K. , Kato, E. , Kautz, M. , Lienert, S. , Lombardozzi, D. , Nabel, J. E. M. S. , Tian, H. , Wiltshire, A. J. , Zhu, D. , Smith, W. K. , & Richardson, A. D. (2018). Widespread seasonal compensation effects of spring warming on northern plant productivity. Nature, 562(7725), 110. 30283105 10.1038/s41586-018-0555-7

[gbc21045-bib-0017] Butterfield, Z. , Buermann, W. , & Keppel‐Aleks, G. (2020). Satellite observations reveal seasonal redistribution of northern ecosystem productivity in response to interannual climate variability. Remote Sensing of Environment, 242, 111755.

[gbc21045-bib-0018] Byrne, B. , Jones, D. B. A. , Strong, K. , Polavarapu, S. M. , Harper, A. B. , Baker, D. F. , & Maksyutov, S. (2019). On what scales can GOSAT flux inversions constrain anomalies in terrestrial ecosystems? Atmospheric Chemistry and Physics, 19(20), 13,017–13,035. 10.5194/acp-19-13017-2019

[gbc21045-bib-0019] Byrne, B. , Jones, D. B. A. , Strong, K. , Zeng, Z.‐C. , Deng, F. , & Liu, J. (2017). Sensitivity of CO_2_ surface flux constraints to observational coverage. Journal of Geophysical Research: Atmospheres, 112, 6672–6694. 10.1002/2016JD026164

[gbc21045-bib-0020] Byrne, B. , Liu, J. , Lee, M. , Baker, I. T. , Bowman, K. W. , Deutscher, N. M. , Feist, D. G. , Griffith, D. W. T. , Iraci, L. T. , Kiel, M. , Kimball, J. , Miller, C. E. , Morino, I. , Parazoo, N. C. , Petri, C. , Roehl, C. M. , Sha, M. , Strong, K. , Velazco, V. A. , Wennberg, P. O. , & Wunch, D. (2020). Improved constraints on northern extratropical CO_2_ fluxes obtained by combining surface‐based and space‐based atmospheric CO_2_ measurements. Journal of Geophysical Research: Atmospheres, 125, e2019JD032029 10.1029/2019JD032029

[gbc21045-bib-0021] Byrne, B. , Wunch, D. , Jones, D. B. A. , Strong, K. , Deng, F. , Baker, I. , Köhler, P. , Frankenberg, C. , Joiner, J. , Arora, V. K. , Badawy, B. , Harper, A. B. , Warneke, T. , Petri, C. , Kivi, R. , & Roehl, C. M. (2018). Evaluating GPP and respiration estimates over northern midlatitude ecosystems using solar‐induced fluorescence and atmospheric CO_2_ measurements. Journal of Geophysical Research: Biogeosciences, 123, 2976–2997. 10.1029/2018JG004472

[gbc21045-bib-0022] Chevallier, F. (2013). On the parallelization of atmospheric inversions of CO_2_ surface fluxes within a variational framework. Geoscientific Model Development, 6(3), 783–790. 10.5194/gmd-6-783-2013

[gbc21045-bib-0023] Chevallier, F. , Ciais, P. , Conway, T. J. , Aalto, T. , Anderson, B. E. , Bousquet, P. , Brunke, E. G. , Ciattaglia, L. , Esaki, Y. , Fröhlich, M. , Gomez, A. , Gomez Pelaez, A. J. , Haszpra, L. , Krummel, P. B. , Langenfelds, R. L. , Leuenberger, M. , Machida, T. , Maignan, F. , Matsueda, H. , Morguí, J. A. , Mukai, H. , Nakazawa, T. , Peylin, P. , Ramonet, M. , Rivier, L. , Sawa, Y. , Schmidt, M. , Steele, L. P. , Vay, S. A. , Vermeulen, A. T. , Wofsy, S. , & Worthy, D. (2010). CO_2_ surface fluxes at grid point scale estimated from a global 21 year reanalysis of atmospheric measurements. Journal of Geophysical Research, 115, D21307 10.1029/2010JD013887

[gbc21045-bib-0024] Chevallier, F. , Fisher, M. , Peylin, P. , Serrar, S. , Bousquet, P. , Bréon, F.‐M. , Chédin, A. , & Ciais, P. (2005). Inferring CO_2_ sources and sinks from satellite observations: Method and application to TOVS data. Journal of Geophysical Research, 110, D24309 10.1029/2005JD006390

[gbc21045-bib-0025] Cook, B. I. , Ault, T. R. , & Smerdon, J. E. (2015). Unprecedented 21st century drought risk in the american southwest and central plains. Science Advances, 1(1), e1400082. 26601131 10.1126/sciadv.1400082 PMC4644081

[gbc21045-bib-0026] Cox, P. M. , Pearson, D. , Booth, B. B. , Friedlingstein, P. , Huntingford, C. , Jones, C. D. , & Luke, C. M. (2013). Sensitivity of tropical carbon to climate change constrained by carbon dioxide variability. Nature, 494(7437), 341–344. 23389447 10.1038/nature11882

[gbc21045-bib-0027] Desai, A. (2016a). (1995‐2014) FLUXNET2015 US‐PFa Park Falls/WLEF, Dataset. 10.18140/FLX/1440089

[gbc21045-bib-0028] Desai, A. (2016b). (1999‐2014) FLUXNET2015 US‐WCr Willow Creek, Dataset. 10.18140/FLX/1440095

[gbc21045-bib-0029] Desai, A. (2016c). (2000‐2014) FLUXNET2015 US‐Los Lost Creek, Dataset. 10.18140/FLX/1440076

[gbc21045-bib-0030] Flechtner, F. , Morton, P. , Watkins, M. , & Webb, F. (2014). Status of the GRACE follow‐on mission, Gravity, geoid and height systems (pp. 117–121): Springer.

[gbc21045-bib-0031] Frankenberg, C. , Fisher, J. B. , Worden, J. , Badgley, G. , Saatchi, S. S. , Lee, J.‐E. , Toon, G. C. , Butz, A. , Jung, M. , Kuze, A. , & Yokota, T. (2011). New global observations of the terrestrial carbon cycle from GOSAT: Patterns of plant fluorescence with gross primary productivity. Geophysical Research Letters, 38, L17706 10.1029/2011GL048738

[gbc21045-bib-0032] Fu, Y. S. H. , Campioli, M. , Vitasse, Y. , De Boeck, H. J. , Van den Berge, J. , AbdElgawad, H. , Asard, H. , Piao, S. , Deckmyn, G. , & Janssens, I. A. (2014). Variation in leaf flushing date influences autumnal senescence and next year's flushing date in two temperate tree species. Proceedings of the National Academy of Sciences, 111(20), 7355–7360. 10.1073/pnas.1321727111 PMC4034254 24799708

[gbc21045-bib-0033] Fu, Z. , Dong, J. , Zhou, Y. , Stoy, P. C. , & Niu, S. (2017). Long term trend and interannual variability of land carbon uptake—The attribution and processes. Environmental Research Letters, 12(1), 014018.

[gbc21045-bib-0034] Gelaro, R. , McCarty, W. , Suárez, M. J. , Todling, R. , Molod, A. , Takacs, L. , Randles, C. A. , Darmenov, A. , Bosilovich, M. G. , Reichle, R. , Wargan, K. , Coy, L. , Cullather, R. , Draper, C. , Akella, S. , Buchard, V. , Conaty, A. , da Silva, A. M. , Gu, W. , Kim, G.‐K. , Koster, R. , Lucchesi, R. , Merkova, D. , Nielsen, J. E. , Partyka, G. , Pawson, S. , Putman, W. , Rienecker, M. , Schubert, S. D. , Sienkiewicz, M. , & Zhao, B. (2017). The Modern‐Era Retrospective Analysis for Research and Applications, Version 2 (MERRA‐2). Journal of Climate, 30(14), 5419–5454. 32020988 10.1175/JCLI-D-16-0758.1 PMC6999672

[gbc21045-bib-0035] Goldstein, A. H. (2016). (1997‐2007) FLUXNET2015 US‐Blo Blodgett Forest, Dataset. 10.18140/FLX/1440068

[gbc21045-bib-0036] Golub, G. H. , & Reinsch, C. (1971). Singular value decomposition and least squares solutions, Linear Algebra (pp. 134–151). Springer.

[gbc21045-bib-0037] Gough, C. , Bohrer, G. , & Curtis, P. (2016a). (2000–2014) FLUXNET2015 US‐UMB Univ. of Mich. Biological Station, Dataset. 10.18140/FLX/1440093

[gbc21045-bib-0038] Gough, C. , Bohrer, G. , & Curtis, P. (2016b). (2007–2014) FLUXNET2015 US‐UMd UMBS Disturbance, Dataset. 10.18140/FLX/1440101

[gbc21045-bib-0039] Guerlet, S. , Basu, S. , Butz, A. , Krol, M. , Hahne, P. , Houweling, S. , Hasekamp, O. P. , & Aben, I. (2013). Reduced carbon uptake during the 2010 Northern Hemisphere summer from GOSAT. Geophysical Research Letters, 40, 2378–2383. 10.1002/grl.50402

[gbc21045-bib-0040] Hu, L. , Andrews, A. E. , Thoning, K. W. , Sweeney, C. , Miller, J. B. , Michalak, A. M. , Dlugokencky, E. , Tans, P. P. , Shiga, Y. P. , Mountain, M. , Nehrkorn, T. , Montzka, S. A. , McKain, K. , Kofler, J. , Trudeau, M. , Michel, S. E. , Biraud, S. C. , Fischer, M. L. , Worthy, D. E. J. , Vaughn, B. H. , White, J. W. C. , Yadav, V. , Basu, S. , & van der Velde, I. R. (2019). Enhanced North American carbon uptake associated with El Niño. Science Advances, 5(6), eaaw0076. 31183402 10.1126/sciadv.aaw0076 PMC6551193

[gbc21045-bib-0041] Huang, L. , He, B. , Chen, A. , Wang, H. , Liu, J. , Lű, A. , & Chen, Z. (2016). Drought dominates the interannual variability in global terrestrial net primary production by controlling semi‐arid ecosystems. Scientific Reports, 6, 24639. 27091439 10.1038/srep24639 PMC4835775

[gbc21045-bib-0042] Huete, A. , Didan, K. , Miura, T. , Rodriguez, E. P. , Gao, X. , & Ferreira, L. G. (2002). Overview of the radiometric and biophysical performance of the MODIS vegetation indices. Remote Sensing of Environment, 83(1–2), 195–213.

[gbc21045-bib-0043] Huntington, T. G. , Weiskel, P. K. , Wolock, D. M. , & McCabe, G. J. (2018). A new indicator framework for quantifying the intensity of the terrestrial water cycle. Journal of Hydrology, 559, 361–372.

[gbc21045-bib-0044] Huntzinger, D. N. , Chaterjee, A. , Moore, D. J. P. , Ohrel, S. , Poulter, A. P. , Walker, A. P. , Jacobson, A. R. , Arain, M. A. , Ciais, P. , Fisher, J. B. , Hayes, D. J. , Huang, M. , Huang, S. , Ito, A. , Jain, A. K. , Lei, H. , Lu, C. , Maignan, F. , Mao, J. , Parazoo, N. C. , Peng, C. , Peng, S. , Poulter, B. , Ricciuto, D. M. , Tian, H. , Shi, X. , Wang, W. , Zeng, N. , Zhao, F. , Zhu, Q. , Yang, J. , & Tao, B. (2018). Chapter 19: Future of the North American carbon cycle, Second State of the Carbon Cycle Report (SOCCR2): A Sustained Assessment Report (pp. 760–809). Washington DC, USA: US Global Change Research Program 10.7930/SOCCR2.2018.Ch19

[gbc21045-bib-0045] Huntzinger, D. N. , Schwalm, C. , Michalak, A. M. , Schaefer, K. , King, A. W. , Wei, Y. , Jacobson, A. , Liu, S. , Cook, R. B. , Post, W. M. , Berthier, G. , Hayes, D. , Huang, M. , Ito, A. , Lei, H. , Lu, C. , Mao, J. , Peng, C. H. , Peng, S. , Poulter, B. , Riccuito, D. , Shi, X. , Tian, H. , Wang, W. , Zeng, N. , Zhao, F. , & Zhu, Q. (2013). The North American carbon program multi‐scale synthesis and terrestrial model intercomparison project Part 1: Overview and experimental design. Geoscientific Model Development, 6(6), 2121–2133. 10.5194/gmd-6-2121-2013

[gbc21045-bib-0046] Huntzinger, D. N. , Schwalm, C. R. , Wei, Y. , Cook, R. B. , Michalak, A. M. , Schaefer, K. , Jacobson, A. R. , Arain, M. A. , Ciais, P. , & Fisher, J. B. (2018). NACP MsTMIP: Global 0.5‐deg Model Outputs in Standard Format (Version 1.0). Oak Ridge, Tennessee, USA: ORNL DAAC. 10.3334/ORNLDAAC/1225

[gbc21045-bib-0047] Ishizawa, M. , Mabuchi, K. , Shirai, T. , Inoue, M. , Morino, I. , Uchino, O. , Yoshida, Y. , Belikov, D. , & Maksyutov, S. (2016). Inter‐annual variability of summertime CO_2_ exchange in Northern Eurasia inferred from GOSAT XCO_2_ . Environmental Research Letters, 11(10), 105001.

[gbc21045-bib-0048] Joiner, J. , Guanter, L. , Lindstrot, R. , Voigt, M. , Vasilkov, A. P. , Middleton, E. M. , Huemmrich, K. F. , Yoshida, Y. , & Frankenberg, C. (2013). Global monitoring of terrestrial chlorophyll fluorescence from moderate spectral resolution near‐infrared satellite measurements: Methodology, simulations, and application to GOME‐2. Atmospheric Measurement Techniques, 6(2), 2803–2823. 10.5194/amt-6-2803-2013

[gbc21045-bib-0049] Joiner, J. , Yoshida, Y. , Guanter, L. , & Middleton, E. M. (2016). New methods for the retrieval of chlorophyll red fluorescence from hyperspectral satellite instruments: Simulations and application to GOME‐2 and SCIAMACHY. Atmospheric Measurement Techniques, 9(8), 3939–3967. 10.5194/amt-9-3939-2016

[gbc21045-bib-0050] Joiner, J. , Yoshida, Y. , Vasilkov, A. P. , Middleton, E. M. , et al. (2011). First observations of global and seasonal terrestrial chlorophyll fluorescence from space. Biogeosciences, 8(3), 637–651.

[gbc21045-bib-0051] Joiner, J. , Yoshida, Y. , Zhang, Y. , Duveiller, G. , Jung, M. , Lyapustin, A. , Wang, Y. , & Tucker, C. (2018). Estimation of terrestrial global gross primary production (GPP) with satellite data‐driven models and eddy covariance flux data. Remote Sensing, 10(9), 1346.

[gbc21045-bib-0052] Jung, M. , Reichstein, M. , Schwalm, C. R. , Huntingford, C. , Sitch, S. , Ahlström, A. , Arneth, A. , Camps‐Valls, G. , Ciais, P. , Friedlingstein, P. , Gans, F. , Ichii, K. , Jain, A. K. , Kato, E. , Papale, D. , Poulter, B. , Raduly, B. , Rdenbeck, C. , Tramontana, G. , Viovy, N. , Wang, Y.‐P. , Weber, U. , Zaehle, S. , & Zeng, N. (2017). Compensatory water effects link yearly global land CO_2_ sink changes to temperature. Nature, 541(7638), 516–520. 28092919 10.1038/nature20780

[gbc21045-bib-0053] Jung, M. , Schwalm, C. , Migliavacca, M. , Walther, S. , Camps‐Valls, G. , Koirala, S. , Anthoni, P. , Besnard, S. , Bodesheim, P. , Carvalhais, N. , Chevallier, F. , Gans, F. , Goll, D. S. , Haverd, V. , Köhler, P. , Ichii, K. , Jain, A. K. , Liu, J. , Lombardozzi, D. , Nabel, J. E. M. S. , Nelson, J. A. , O'Sullivan, M. , Pallandt, M. , Papale, D. , Peters, W. , Pongratz, J. , Rödenbeck, C. , Sitch, S. , Tramontana, G. , Walker, A. , Weber, U. , & Reichstein, M. (2020). Scaling carbon fluxes from eddy covariance sites to globe: Synthesis and evaluation of the FLUXCOM approach. Biogeosciences, 17(5), 1343–1365. 10.5194/bg-17-1343-2020

[gbc21045-bib-0054] Keenan, T. F. , & Richardson, A. D. (2015). The timing of autumn senescence is affected by the timing of spring phenology: Implications for predictive models. Global Change Biology, 21(7), 2634–2641. 25662890 10.1111/gcb.12890

[gbc21045-bib-0055] Lam, E. (2004). Controlled cell death, plant survival and development. Nature Reviews Molecular Cell Biology, 5(4), 305. 15071555 10.1038/nrm1358

[gbc21045-bib-0056] Landerer, F. W. , & Swenson, S. C. (2012). Accuracy of scaled GRACE terrestrial water storage estimates. Water Resources Research, 48, W04531 10.1029/2011WR011453

[gbc21045-bib-0057] Liu, Z. , Ballantyne, A. P. , Poulter, B. , Anderegg, W. R. , Li, W. , Bastos, A. , & Ciais, P. (2018). Precipitation thresholds regulate net carbon exchange at the continental scale. Nature Communications, 9(1), 3596. 10.1038/s41467-018-05948-1 PMC6125429 30185789

[gbc21045-bib-0058] Liu, J. , Baskarran, L. , Bowman, K. , Schimel, D. , Bloom, A. A. , Parazoo, N. C. , Oda, T. , Carroll, D. , Menemenlis, D. , Joiner, J. , Commane, R. , Daube, B. , Gatii, L. V. , McKain, K. , Miller, J. , Stephens, B. B. , Sweeney, C. , & Wofsy, S. (2020). Carbon monitoring system flux net biosphere exchange 2020 (CMS‐Flux NBE 2020). Earth System Science Data Discussions, 2020, 1–53. 10.5194/essd-2020-123

[gbc21045-bib-0059] Liu, J. , Bowman, K. , Parazoo, N. C. , Bloom, A. A. , Wunch, D. , Jiang, Z. , Gurney, K. R. , & Schimel, D. (2018). Detecting drought impact on terrestrial biosphere carbon fluxes over contiguous US with satellite observations. Environmental Research Letters, 13(9), 095003.

[gbc21045-bib-0060] Liu, J. , Bowman, K. W. , Schimel, D. S. , Parazoo, N. C. , Jiang, Z. , Lee, M. , Bloom, A. A. , Wunch, D. , Frankenberg, C. , Sun, Y. , O'Dell, C. W. , Gurney, K. R. , Menemenlis, D. , Gierach, M. , Crisp, D. , & Eldering, A. (2017). Contrasting carbon cycle responses of the tropical continents to the 2015–2016 El Niño. Science, 358, 6360 10.1126/science.aam5690 29026011

[gbc21045-bib-0061] Liu, Y. Y. , Dorigo, W. A. , Parinussa, R. , de Jeu, R. A. , Wagner, W. , McCabe, M. F. , Evans, J. P. , & Van Dijk, A. (2012). Trend‐preserving blending of passive and active microwave soil moisture retrievals. Remote Sensing of Environment, 123, 280–297.

[gbc21045-bib-0062] Liu, Z. , Kimball, J. S. , Parazoo, N. C. , Ballantyne, A. P. , Wang, W. J. , Madani, N. , Pan, C. G. , Watts, J. D. , Reichle, R. H. , Sonnentag, O. , Marsh, P. , Hurkuck, M. , Quinton, W. L. , Zona, D. , Ueyama, M. , Kobayashi, H. , & Euskirchen, E. A. S. (2020). Increased high‐latitude photosynthetic carbon gain offset by respiration carbon loss during an anomalous warm winter to spring transition. Global Change Biology, 26(2), 682–696. 31596019 10.1111/gcb.14863

[gbc21045-bib-0063] Liu, Y. Y. , Parinussa, R. M. , Dorigo, W. A. , De Jeu, R. A. , Wagner, W. , Van Dijk, A. I. , McCabe, M. F. , & Evans, J. P. (2011). Developing an improved soil moisture dataset by blending passive and active microwave satellite‐based retrievals. Hydrology and Earth System Sciences, 15(2), 425–436.

[gbc21045-bib-0064] Massman, B. (2016). (2004‐2014) FLUXNET2015 US‐GLE GLEES, Dataset. 10.18140/FLX/1440069

[gbc21045-bib-0065] Niu, S. , Fu, Z. , Luo, Y. , Stoy, P. C. , Keenan, T. F. , Poulter, B. , Zhang, L. , Piao, S. , Zhou, X. , Zheng, H. , Han, J. , Wang, Q. , & Yu, G. (2017). Interannual variability of ecosystem carbon exchange: From observation to prediction. Global Ecology and Biogeography, 26(11), 1225–1237.

[gbc21045-bib-0066] Novick, K. , & Phillips, R. (2016). (1999‐2014) FLUXNET2015 US‐MMS Morgan Monroe State Forest, Dataset. 10.18140/FLX/1440083

[gbc21045-bib-0067] Papageorgiou, G. C. , & Govindjee (2007). Chlorophyll a fluorescence: A signature of photosynthesis (Vol. 19).Springer Science & Business Media.

[gbc21045-bib-0068] Papagiannopoulou, C. , Miralles, D. , Dorigo, W. A. , Verhoest, N. , Depoorter, M. , & Waegeman, W. (2017). Vegetation anomalies caused by antecedent precipitation in most of the world. Environmental Research Letters, 12(7), 074016.

[gbc21045-bib-0069] Parazoo, N. C. , Barnes, E. , Worden, J. , Harper, A. B. , Bowman, K. B. , Frankenberg, C. , Wolf, S. , Litvak, M. , & Keenan, T. F. (2015). Influence of ENSO and the NAO on terrestrial carbon uptake in the Texas‐Northern Mexico region. Global Biogeochemical Cycles, 29, 1247–1265. 10.1002/2015GB005125

[gbc21045-bib-0070] Parazoo, N. C. , Bowman, K. , Fisher, J. B. , Frankenberg, C. , Jones, D. , Cescatti, A. , Pérez‐Priego, O. , Wohlfahrt, G. , & Montagnani, L. (2014). Terrestrial gross primary production inferred from satellite fluorescence and vegetation models. Global Change Biology, 20(10), 3103–3121. 24909755 10.1111/gcb.12652

[gbc21045-bib-0071] Parida, B. R. , & Buermann, W. (2014). Increasing summer drying in north american ecosystems in response to longer nonfrozen periods. Geophysical Research Letters, 41, 5476–5483. 10.1002/2014GL060495

[gbc21045-bib-0072] Pastorello, G. , Trotta, C. , Canfora, E. , Chu, H. , Christianson, D. , Cheah, Y.‐W. , Poindexter, C. , Chen, J. , Elbashandy, A. , Humphrey, M. , Isaac, P. , Polidori, D. , Ribeca, A. , van Ingen, C. , Zhang, L. , Amiro, B. , Ammann, C. , Arain, M. A. , Ardö, J. , Arkebauer, T. , Arndt, S. K. , Arriga, N. , Aubinet, M. , Aurela, M. , Baldocchi, D. , Barr, A. , Beamesderfer, E. , Marchesini, L. B. , Bergeron, O. , Beringer, J. , Bernhofer, C. , Berveiller, D. , Billesbach, D. , Black, T. A. , Blanken, P. D. , Bohrer, G. , Boike, J. , Bolstad, P. V. , Bonal, D. , Bonnefond, J.‐M. , Bowling, D. R. , Bracho, R. , Brodeur, J. , Brümmer, C. , Buchmann, N. , Burban, B. , Burns, S. P. , Buysse, P. , Cale, P. , Cavagna, M. , Cellier, P. , Chen, S. , Chini, I. , Christensen, T. R. , Cleverly, J. , Collalti, A. , Consalvo, C. , Cook, B. D. , Cook, D. , Coursolle, C. , Cremonese, E. , Curtis, P. S. , D'Andrea, E. , da Rocha, H. , Dai, X. , Davis, K. J. , De Cinti, B. , de Grandcourt, A. , De Ligne, A. , De Oliveira, R. C. , Delpierre, N. , Desai, A. R. , Di Bella, C. M. , di Tommasi, P. , Dolman, H. , Domingo, F. , Dong, G. , Dore, S. , Duce, P. , Dufrêne, E. , Dunn, A. , Dušek, J. A. , Eamus, D. , Eichelmann, U. , ElKhidir, H. A. M. , Eugster, W. , Ewenz, C. M. , Ewers, B. , Famulari, D. , Fares, S. , Feigenwinter, I. , Feitz, A. , Fensholt, R. , Filippa, G. , Fischer, M. , Frank, J. , Galvagno, M. , Gharun, M. , Gianelle, D. , Gielen, B. , Gioli, B. , Gitelson, A. , Goded, I. , Goeckede, M. , Goldstein, A. H. , Gough, C. M. , Goulden, M. L. , Graf, A. , Griebel, A. , Gruening, C. , Grünwald, T. , Hammerle, A. , Han, S. , Han, X. , Hansen, B. U. , Hanson, C. , Hatakka, J. , He, Y. , Hehn, M. , Heinesch, B. , Hinko‐Najera, N. , Hörtnagl, L. , Hutley, L. , Ibrom, A. , Ikawa, H. , Jackowicz‐Korczynski, M. , Janou, D. , Jans, W. , Jassal, R. , Jiang, S. , Kato, T. , Khomik, M. , Klatt, J. , Knohl, A. , Knox, S. , Kobayashi, H. , Koerber, G. , Kolle, O. , Kosugi, Y. , Kotani, A. , Kowalski, A. , Kruijt, B. , Kurbatova, J. , Kutsch, W. L. , Kwon, H. , Launiainen, S. , Laurila, T. , Law, B. , Leuning, R. , Li, Y. , Liddell, M. , Limousin, J.‐M. , Lion, M. , Liska, A. J. , Lohila, A. , López‐Ballesteros, A. , López‐Blanco, E. A. , Loubet, B. , Loustau, D. , Lucas‐Moffat, A. , Lüers, J. , Ma, S. , Macfarlane, C. , Magliulo, V. , Maier, R. , Mammarella, I. , Manca, G. , Marcolla, B. , Margolis, H. A. , Marras, S. , Massman, W. , Mastepanov, M. , Matamala, R. , Matthes, J. H. , Mazzenga, F. , McCaughey, H. , McHugh, I. , McMillan, A. M. S. , Merbold, L. , Meyer, W. , Meyers, T. , Miller, S. D. , Minerbi, S. , Moderow, U. , Monson, R. K. , Montagnani, L. , Moore, C. E. , Moors, E. , Moreaux, V. , Moureaux, C. , Munger, J. W. , Nakai, T. , Neirynck, J. , Nesic, Z. , Nicolini, G. , Noormets, A. , Northwood, M. , Nosetto, M. , Nouvellon, Y. , Novick, K. , Oechel, W. , Olesen, J. A. B. E. , Ourcival, J.‐M. , Papuga, S. A. , Parmentier, F.‐J. , Paul‐Limoges, E. , Pavelka, M. , Peichl, M. , Pendall, E. , Phillips, R. P. , Pilegaard, K. , Pirk, N. , Posse, G. , Powell, T. , Prasse, H. , Prober, S. M. , Rambal, S. , Rannik, A. , Raz‐Yaseef, N. , Reed, D. , de Dios, V. R. , Restrepo‐Coupe, N. , Reverter, B. R. , Roland, M. , Sabbatini, S. , Sachs, T. , Saleska, S. R. , Sánchez‐Cañete, E. P. , Sanchez‐Mejia, Z. M. , Schmid, H. P. , Schmidt, M. , Schneider, K. , Schrader, F. , Schroder, I. , Scott, R. L. , Sedlák, P. , Serrano‐Ortíz, P. A. , Shao, C. , Shi, P. , Shironya, I. , Siebicke, L. , Šigut, L. , Silberstein, R. , Sirca, C. , Spano, D. , Steinbrecher, R. , Stevens, R. M. , Sturtevant, C. , Suyker, A. , Tagesson, T. , Takanashi, S. , Tang, Y. , Tapper, N. , Thom, J. , Tiedemann, F. , Tomassucci, M. , Tuovinen, J.‐P. , Urbanski, S. , Valentini, R. , van der Molen, M. , van Gorsel, E. , van Huissteden, K. , Varlagin, A. , Verfaillie, J. , Vesala, T. , Vincke, C. , Vitale, D. , Vygodskaya, N. , Walker, J. P. , Walter‐Shea, E. , Wang, H. , Weber, R. , Westermann, S. , Wille, C. , Wofsy, S. , Wohlfahrt, G. , Wolf, S. , Woodgate, W. , Li, Y. , Zampedri, R. , Zhang, J. , Zhou, G. , Zona, D. , Agarwal, D. , Biraud, S. , Torn, M. , & Papale, D. (2020). The FLUXNET2015 dataset and the ONEFlux processing pipeline for eddy covariance data. Scientific Data, 7(1), 225 10.1038/s41597-020-0534-3 32647314 PMC7347557

[gbc21045-bib-0073] Peters, W. , Jacobson, A. R. , Sweeney, C. , Andrews, A. E. , Conway, T. J. , Masarie, K. , Miller, J. B. , Bruhwiler, L. M. P. , Pétron, G. , Hirsch, A. I. , Worthy, D. E. J. , van der Werf, G. R. , Randerson, J. T. , Wennberg, P. O. , Krol, M. C. , & Tans, P. P. (2007). An atmospheric perspective on North American carbon dioxide exchange: CarbonTracker. Proceedings of the National Academy of Sciences of the United States of America, 104(48), 18,925–18,930. 10.1073/pnas.0708986104 PMC2141884 18045791

[gbc21045-bib-0074] Poulter, B. , Frank, D. , Ciais, P. , Myneni, R. B. , Andela, N. , Bi, J. , Broquet, G. , Canadell, J. G. , Chevallier, F. , Liu, Y. Y. , Running, S. W. , Sitch, S. , & van der Werf, G. R. (2014). Contribution of semi‐arid ecosystems to interannual variability of the global carbon cycle. Nature, 509(7502), 600. 24847888 10.1038/nature13376

[gbc21045-bib-0075] Reich, P. B. , Walters, M. B. , & Ellsworth, D. S. (1992). Leaf life‐span in relation to leaf, plant, and stand characteristics among diverse ecosystems. Ecological Monographs, 62(3), 365–392.

[gbc21045-bib-0076] Reichle, R. H. , Draper, C. S. , Liu, Q. , Girotto, M. , Mahanama, S. P. , Koster, R. D. , & De Lannoy, G. J. (2017). Assessment of MERRA‐2 land surface hydrology estimates. Journal of Climate, 30(8), 2937–2960.

[gbc21045-bib-0077] Reichle, R. H. , Koster, R. D. , De Lannoy, G. J. M. , Forman, B. A. , Liu, Q. , Mahanama, S. P. , & Touré, A. (2011). Assessment and enhancement of MERRA land surface hydrology estimates. Journal of Climate, 24(24), 6322–6338.

[gbc21045-bib-0078] Remaud, M. , Chevallier, F. , Cozic, A. , Lin, X. , & Bousquet, P. (2018). On the impact of recent developments of the LMDz atmospheric general circulation model on the simulation of CO_2_ transport. Geoscientific Model Development, 11(11), 4489–4513. 10.5194/gmd-11-4489-2018

[gbc21045-bib-0079] Rödenbeck, C. , Zaehle, S. , Keeling, R. , & Heimann, M. (2018). How does the terrestrial carbon exchange respond to inter‐annual climatic variations? A quantification based on atmospheric CO_2_ data. Biogeosciences, 15(8), 2481–2498. 10.5194/bg-15-2481-2018

[gbc21045-bib-0080] Schaaf, C. B. , Gao, F. , Strahler, A. H. , Lucht, W. , Li, X. , Tsang, T. , Strugnell, N. C. , Zhang, X. , Jin, Y. , Muller, J.‐P. , Lewis, P. , Barnsley, M. , Hobson, P. , Disney, M. , Roberts, G. , Dunderdale, M. , Doll, C. , d'Entremont, R. P. , Hu, B. , Liang, S. , Privette, J. L. , & Roy, D. (2002). First operational BRDF, albedo nadir reflectance products from MODIS. Remote Sensing of Environment, 83(1–2), 135–148.

[gbc21045-bib-0081] Schwalm, C. R. , Williams, C. A. , Schaefer, K. , Arneth, A. , Bonal, D. , Buchmann, N. , Chen, J. , Law, B. E. , Lindroth, A. , Luyssaert, S. , Reichstein, M. , & Richardson, A. D. (2010). Assimilation exceeds respiration sensitivity to drought: A FLUXNET synthesis. Global Change Biology, 16(2), 657–670.

[gbc21045-bib-0082] Schwalm, C. R. , Williams, C. A. , Schaefer, K. , Baldocchi, D. , Black, T. A. , Goldstein, A. H. , Law, B. E. , Oechel, W. C. , & Scott, R. L. (2012). Reduction in carbon uptake during turn of the century drought in Western North America. Nature Geoscience, 5(8), 551.

[gbc21045-bib-0083] Scott, R. (2016a). (2004‐2014) FLUXNET2015 US‐SRM Santa Rita Mesquite, Dataset. 10.18140/FLX/1440090

[gbc21045-bib-0084] Scott, R. (2016b). (2004‐2014) FLUXNET2015 US‐Wkg Walnut Gulch Kendall Grasslands, Dataset. 10.18140/FLX/1440096

[gbc21045-bib-0085] Scott, R. (2016c). (2007‐2014) FLUXNET2015 US‐Whs Walnut Gulch Lucky Hills Shrub, Dataset. 10.18140/FLX/1440097

[gbc21045-bib-0086] Scott, R. (2016d). (2008‐2014) FLUXNET2015 US‐SRG Santa Rita Grassland, Dataset. 10.18140/FLX/1440114

[gbc21045-bib-0087] Seager, R. , Ting, M. , Held, I. , Kushnir, Y. , Lu, J. , Vecchi, G. , Huang, H.‐P. , Harnik, N. , Leetmaa, A. , Lau, N.‐C. , Li, C. , Velez, J. , & Naik, N. (2007). Model projections of an imminent transition to a more arid climate in southwestern North America. Science, 316(5828), 1181–1184. 17412920 10.1126/science.1139601

[gbc21045-bib-0088] Shiga, Y. P. , Michalak, A. M. , Fang, Y. , Schaefer, K. , Andrews, A. E. , Huntzinger, D. H. , Schwalm, C. R. , Thoning, K. , & Wei, Y. (2018). Forests dominate the interannual variability of the North American carbon sink. Environmental Research Letters, 13(8), 084015. 10.1088/1748-9326/aad505

[gbc21045-bib-0089] Smith, T. E. L. , Wooster, M. J. , Tattaris, M. , & Griffith, D. W. T. (2011). Absolute accuracy and sensitivity analysis of OP‐FTIR retrievals of CO_2_, CH_4_ and CO over concentrations representative of “clean air” and “polluted plumes”. Atmospheric Measurement Techniques, 4(1), 97–116. 10.5194/amt-4-97-2011

[gbc21045-bib-0090] Sun, Y. , Frankenberg, C. , Wood, J. D. , Schimel, D. S. , Jung, M. , Guanter, L. , Drewry, D. T. , Verma, M. , Porcar‐Castell, A. , Griffis, T. J. , Gu, L. , Magney, T. , Köhler, P. , Evans, B. J. , & Yuen, K. (2017). OCO‐2 advances photosynthesis observation from space via solar‐induced chlorophyll fluorescence. Science, 358(6360), eaam5747. 29026013 10.1126/science.aam5747

[gbc21045-bib-0091] Suyker, A. (2016a). (2001‐2013) FLUXNET2015 US‐Ne1 Mead‐irrigated continuous maize site, Dataset. 10.18140/FLX/1440084

[gbc21045-bib-0092] Suyker, A. (2016b). (2001‐2013) FLUXNET2015 US‐Ne2 Mead‐irrigated maize‐soybean rotation site, Dataset. 10.18140/FLX/1440085

[gbc21045-bib-0093] Suyker, A. (2016c). (2001‐2013) FLUXNET2015 US‐Ne3 Mead‐rainfed maize‐soybean rotation site, Dataset. 10.18140/FLX/1440086

[gbc21045-bib-0094] Tapley, B. D. , Bettadpur, S. , Ries, J. C. , Thompson, P. F. , & Watkins, M. M. (2004). GRACE measurements of mass variability in the Earth system. Science, 305(5683), 503–505. 15273390 10.1126/science.1099192

[gbc21045-bib-0095] Tramontana, G. , Jung, M. , Schwalm, C. R. , Ichii, K. , Camps‐Valls, G. , Ráduly, B. , Reichstein, M. , Arain, M. A. , Cescatti, A. , Kiely, G. , Merbold, L. , Serrano‐Ortiz, P. , Sickert, S. , Wolf, S. , & Papale, D. (2016). Predicting carbon dioxide and energy fluxes across global FLUXNET sites with regression algorithms. Biogeosciences, 13(14), 4291–4313. 10.5194/bg-13-4291-2016

[gbc21045-bib-0096] Vose, R. S. , Easterling, D. R. , Kunkel, K. E. , LeGrande, A. N. , & Wehner, M. F. (2017). Temperature changes in the United States InWuebbles D. J. et al. (Eds.), Climate Science Special Report: Fourth National Climate Assessment, Volume I (pp. 185–206). Washington, DC, USA: U.S. Global Change Research Program 10.7930/J0N29V45

[gbc21045-bib-0097] Wei, Y. , Liu, S. , Huntzinger, D. N. , Michalak, A. M. , Viovy, N. , Post, W. M. , Schwalm, C. R. , Schaefer, K. , Jacobson, A. R. , Lu, C. , Tian, H. , Ricciuto, D. M. , Cook, R. B. , Mao, J. , & Shi, X. (2014). The North American carbon program multi‐scale synthesis and terrestrial model intercomparison project—Part 2: Environmental driver data. Geoscientific Model Development, 7(6), 2875–2893. 10.5194/gmd-7-2875-2014

[gbc21045-bib-0098] Wolf, S. , Keenan, T. F. , Fisher, J. B. , Baldocchi, D. D. , Desai, A. R. , Richardson, A. D. , Scott, R. L. , Law, B. E. , Litvak, M. E. , Brunsell, N. A. , Peters, W. , & van der Laan‐Juijkx, I. T. (2016). Warm spring reduced carbon cycle impact of the 2012 US summer drought. Proceedings of the National Academy of Sciences, 113(21), 5880–5885. 10.1073/pnas.1519620113 PMC4889356 27114518

[gbc21045-bib-0099] Yang, X. , Tang, J. , Mustard, J. F. , Lee, J.‐E. , Rossini, M. , Joiner, J. , Munger, J. W. , Kornfeld, A. , & Richardson, A. D. (2015). Solar‐induced chlorophyll fluorescence that correlates with canopy photosynthesis on diurnal and seasonal scales in a temperate deciduous forest. Geophysical Research Letters, 42, 2977–2987. 10.1002/2015GL063201

[gbc21045-bib-0100] Yin, Y. , Byrne, B. , Liu, J. , Wennberg, P. O. , Davis, K. J. , Magney, T. , Köhler, P. , He, L. , Jeyaram, R. , Humphrey, V. , Gerken, T. , Feng, S. , Digangi, J. P. , & Frankenberg, C. (2020). Cropland carbon uptake delayed and reduced by 2019 midwest floods. AGU Advances, 1(1), e2019AV000140.

[gbc21045-bib-0101] Zhang, X. , Gurney, K. R. , Peylin, P. , Chevallier, F. , Law, R. M. , Patra, P. K. , Rayner, P. J. , Röedenbeck, C. , & Krol, M. (2013). On the variation of regional CO_2_ exchange over temperate and boreal North America. Global Biogeochemical Cycles, 27, 991–1000. 10.1002/gbc.20091

