# Supplementary material for: Contrasting Regional Carbon Cycle Responses to Seasonal Climate Anomalies Across the East‐West Divide of Temperate North America
Source: Global Biogeochem Cycles. 2020 Nov 13;34(11):e2020GB006598. doi: 10.1029/2020GB006598 (PMC7685151; doi:10.1029/2020GB006598)
Supplement: Supplementary file 1 — Supporting Information S1 [file GBC-34-e2020GB006598-s001.pdf]

Supporting Information for "Contrasting regional carbon cycle responses to seasonal climate anomalies across the east-west divide of temperate North America"

B. Byrne[1], J. Liu[1,2], A. A. Bloom[1], K. W. Bowman[1,3], Z. Butterfield[4],

J. Joiner[5], T. F. Keenan[6,7], G. Keppel-Aleks[4], N. C. Parazoo[1], and Y. Yin[2]

[1]Jet Propulsion Laboratory, California Institute of Technology, CA, USA

[2]Division of Geological and Planetary Sciences, California Institute of Technology, Pasadena, CA, USA

[3]Joint Institute for Regional Earth System Science and Engineering, University of California, Los Angeles, USA

[4]Department of Climate and Space Sciences and Engineering, University of Michigan, Ann Arbor, MI, USA

[5]Laboratory of Atmospheric Chemistry and Dynamics, NASA Goddard Space Flight Center, Greenbelt, MD 20771, USA

[6]Earth and Environmental Sciences Area, Lawrence Berkeley National Laboratory, Berkeley, California, USA

[7]Department of Environmental Science, Policy and Management, University of California, Berkeley, Berkeley, California, USA

Contents of this file

1. Text S1
2. Figures S1 to S9
3. Table S1

Text S1. Flux inversion NEE

CT2017 (Peters et al. (2007), with updates documented at https://www.esrl.noaa.gov/gmd/ccgg/carbontracker/) optimizes NEE by assimilating flask and in situ CO_2 measurements. It employs an ensemble Kalman filter approach to assimilate CO_2 with atmospheric chemical transport simulated by the TM5 offline atmospheric model (Krol et al., 2005). For CT2017, TM5 is driven by ERA-Interim assimilated meteorology from the European Centre for Medium-Range Weather Forecasts (ECMWF), with a horizontal resolution of $3^\circ \times 2^\circ$ globally and $1^\circ \times 1^\circ$ in a nested grid over temperate North America.

CT-L provides estimates of posterior NEE over temperate North America by performing analytic Bayesian inversions assimilating flask and in situ CO_2 measurements (Hu et al., 2019). For these inversions, contributions to the observations from background CO_2 inflow over temperate North America and surface CO_2 emissions from fossil fuel and fire emissions are pre-subtracted from the observations. Footprint sensitivities of the observations to surface fluxes are calculated using the high-resolution Weather Research and Forecasting–Stochastic Time–Inverted Lagrangian Transport (WRF-STILT) model at 10 km spatial resolution over the domain of interest, providing footprints at 1x1 and hourly temporal resolution and represent simulated upwind influences over 10 days before each measurement. An ensemble of 18 flux inversions is performed by varying prior NEE and boundary conditions in the inversion. Here we examine the ensemble mean from CT-L. Monthly NEE fluxes at 1x1 spatial resolution were downloaded from https://doi.org/10.15138/3dw1-5c37.

The CAMS greenhouse gases inversion system (v18r3) (Chevallier et al., 2005, 2010; Chevallier, 2013) assimilates surface air-sample CO_2 measurements from 129 sites over the globe. 4-DVar is employed to optimize day-time and night-time NEE at 8-day temporal resolution of $1.875° \times 3.75°$ model grid. Tracer transport is performed using the Laboratoire de Météorologie Dynamique (LMDz) general circulation model version LMDz6A (Remaud et al., 2018). These data were downloaded from https://atmosphere.copernicus.eu/.

References

Byrne, B., Liu, J., Lee, M., Baker, I. T., Bowman, K. W., Deutscher, N. M., ... Wunch, D. (2020). Improved constraints on northern extratropical CO_2 fluxes obtained by combining surface-based and space-based atmospheric CO_2 measurements. *Journal ofGeophysical Research: Atmospheres*, *125*. doi: 10.1029/2019JD032029

Chevallier, F. (2013). On the parallelization of atmospheric inversions of co_2 surface fluxes within a variational framework. *Geoscientific Model Development*, *6*(3), 783–790. Retrieved from `https://gmd.copernicus.org/articles/6/783/2013/` doi: 10.5194/gmd-6-783-2013

Chevallier, F., Ciais, P., Conway, T., Aalto, T., Anderson, B., Bousquet, P., ... Worthy, D. (2010). CO_2 surface fluxes at grid point scale estimated from a global 21 year reanalysis of atmospheric measurements. *Journal of Geophysical Research: Atmospheres*, *115*(D21).

Chevallier, F., Fisher, M., Peylin, P., Serrar, S., Bousquet, P., Bréon, F.-M., ... Ciais, P. (2005). Inferring CO_2 sources and sinks from satellite observations: Method and application to TOVS data. *Journal of Geophysical Research: Atmospheres*, *110*(D24).

Hu, L., Andrews, A. E., Thoning, K. W., Sweeney, C., Miller, J. B., Michalak, A. M., ... others (2019). Enhanced north american carbon uptake associated with el niño. *Science advances*, *5*(6), eaaw0076.

Krol, M., Houweling, S., Bregman, B., Broek, M., Segers, A., Velthoven, P. v., ... Bergamaschi, P. (2005). The two-way nested global chemistry-transport zoom model tm5: algorithm and applications. *Atmos. Chem. Phys.*, *5*(2), 417–432. doi: 10.5194/acp-5-417-2005

Peters, W., Jacobson, A. R., Sweeney, C., Andrews, A. E., Conway, T. J., Masarie, K., ... others (2007). An atmospheric perspective on North American carbon dioxide exchange: CarbonTracker. *Proc. Natl. Acad. Sci.*, *104*(48), 18925–18930. doi: 10.1073/pnas.0708986104

Remaud, M., Chevallier, F., Cozic, A., Lin, X., & Bousquet, P. (2018). On the impact of recent developments of the lmdz atmospheric general circulation model on the simulation of CO_2 transport. *Geoscientific Model Development*, *11*(11), 4489–4513. Retrieved from https://gmd.copernicus.org/articles/11/4489/2018/ doi: 10.5194/gmd-11-4489-2018

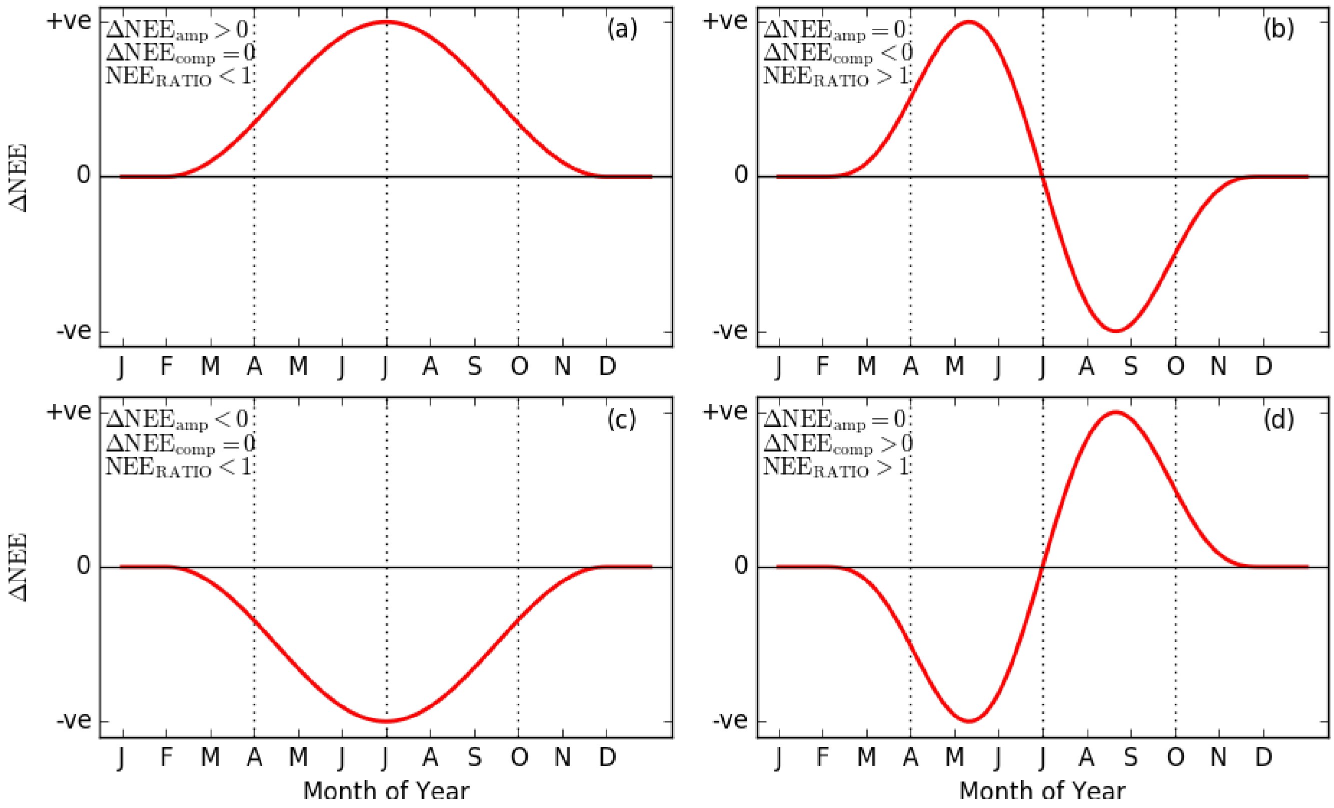

Figure S1. Illustration of amplification and compensation for NEE. (a) Positive amplification with no compensation, (b) no amplification with negative compensation, (c) negative amplification with no compensation, and (d) no amplification with positive amplification. Note that the same scheme is applied to GPP.

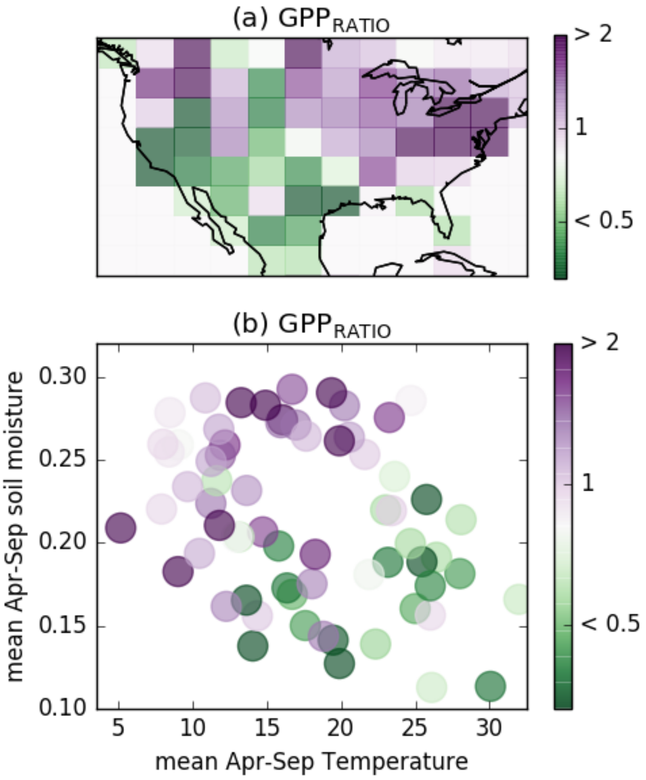

Figure S2. Relative magnitudes of seasonal compensation and amplification. (a) GPP_{RATIO} over 2010–2015 at $4^\circ \times 5^\circ$. (b) GPP_{RATIO} plotted as a function of Apr-Sep mean soil temperature (K) and soil moisture ($m^3\,m^{-3}$).

Table S1. Correlation coefficient (R) between CO_2 flux anomalies and $\Delta T_{Apr-Sep}$, $\Delta M_{Apr-Sep}$, $\Delta P_{Apr-Sep}$, or $\Delta TWS_{Apr-Sep}$. Correlations cover the period 2001–2017 for ΔGPP and 2010–2015 for ΔNEE, except for correlations with ΔTWS which cover 2003–2014 and 2010–2014. For eastern North America, 2012 is excluded for ΔGPP correlations because it is an extreme event and has a large impact on the correlation.

Region	Environ Var	ΔGPP_{amp}	ΔGPP_{comp}	ΔNEE_{amp}	ΔNEE_{comp}
West	$\Delta T_{Apr-Sep}$	-0.71	-0.41	0.63	0.22
West	$\Delta M_{Apr-Sep}$	0.91	0.09	-0.66	-0.13
West	$\Delta P_{Apr-Sep}$	0.78	0.31	-0.47	-0.21
West	$\Delta TWS_{Apr-Sep}$	0.50	0.20	-0.70	-0.19
East	$\Delta T_{Apr-Sep}$	-0.09	-0.81	-0.46	0.89
East	$\Delta M_{Apr-Sep}$	0.72	0.41	0.78	-0.49
East	$\Delta P_{Apr-Sep}$	0.35	0.31	0.48	-0.44
East	$\Delta TWS_{Apr-Sep}$	0.56	0.31	0.81	-0.30

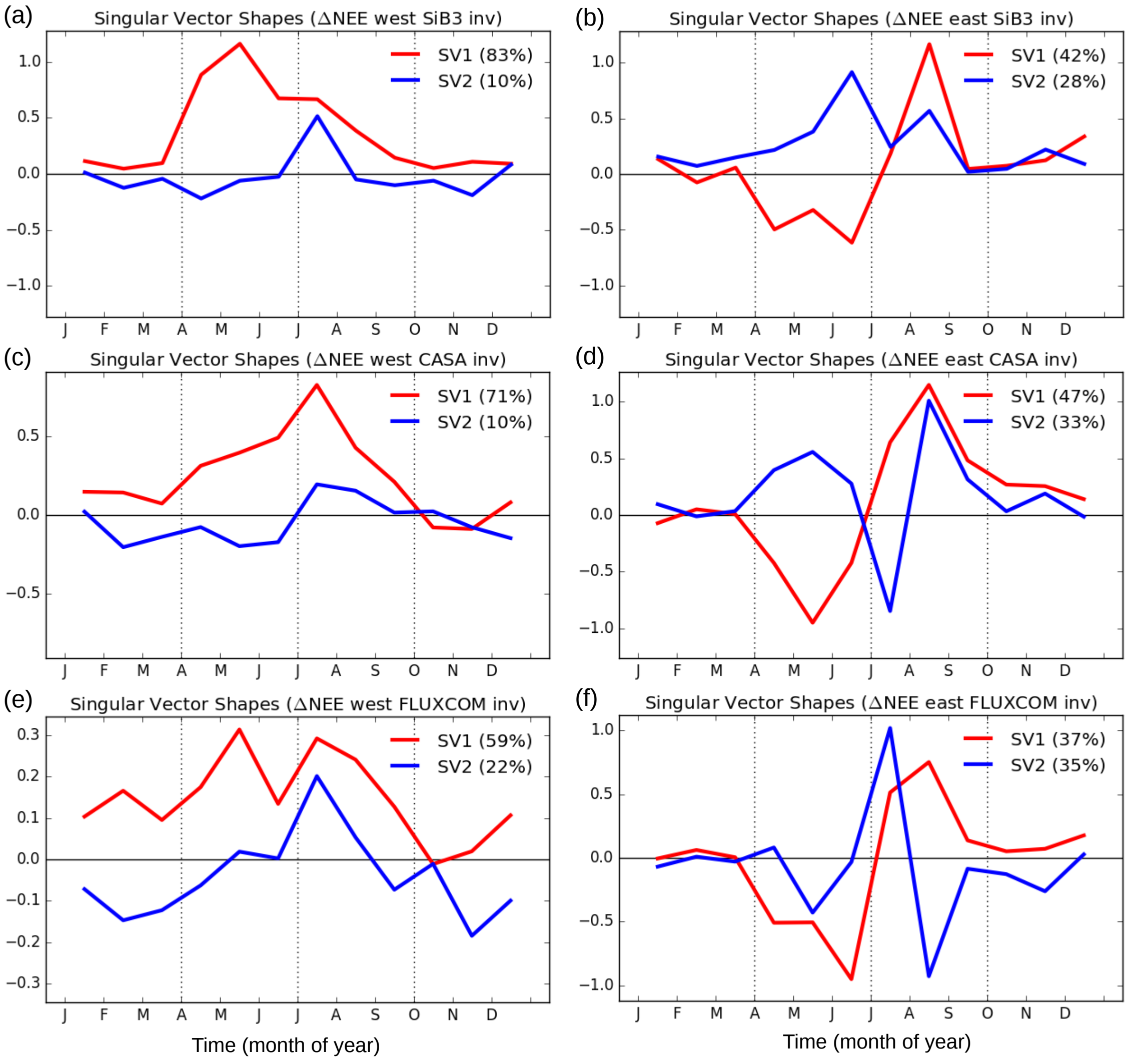

Figure S3. SVD analysis for individual flux inversions from Byrne et al. (2020) for (a) western and (b) eastern North America

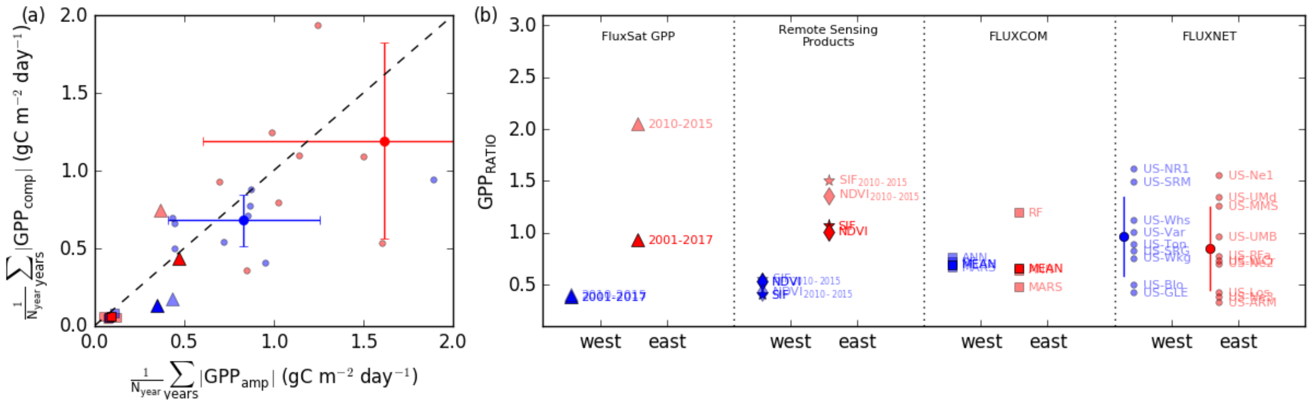

Figure S4. (a) Mean magnitude of GPP compensation versus mean magnitude of GPP amplification across multiple years. (b) GPP_{RATIO} over eastern and western North America for (left-to-right) FluxSat, remotely sensed products (GOME-2 SIF and MODIS NDVI), FLUXCOM GPP, and FLUXNET sites with 6+ years of data within the eastern and western domains. Partially transparent symbols show values over 2010–2015 and solid colors are for the entire time period examined in this study for a given dataset. Note that NDVI and SIF are not shown in panel (a) because they do not have units of GPP.

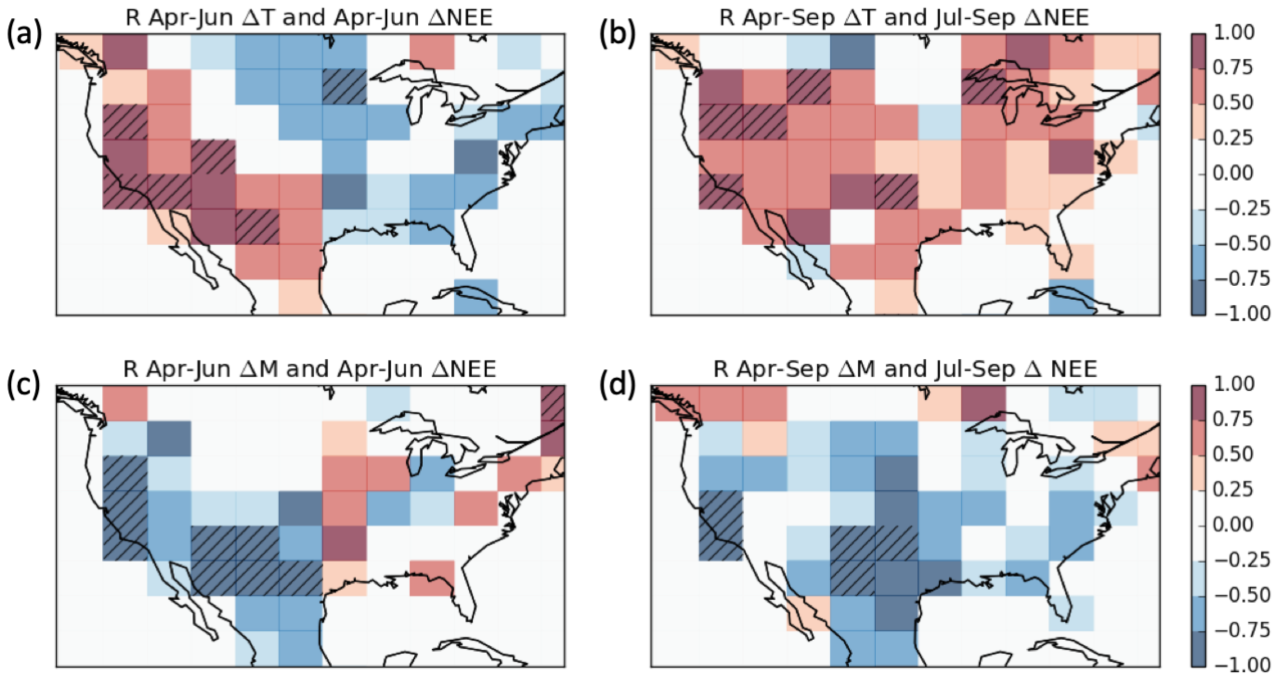

Figure S5. Relationship between ΔNEE and variations in climate. Coefficient of correlation (R) over 2010–2015 for $4^\circ \times 5^\circ$ grid cells between (a) Apr–Jun ΔT and Apr–Jun ΔNEE, (b) Apr–Sep ΔT and Jul–Sep ΔNEE, (c) Apr–Jun ΔM and Apr–Jun ΔNEE and (d) Apr–Sep ΔM and Jul–Sep ΔNEE. Hatching shows grid cells for which $P < 0.05$.

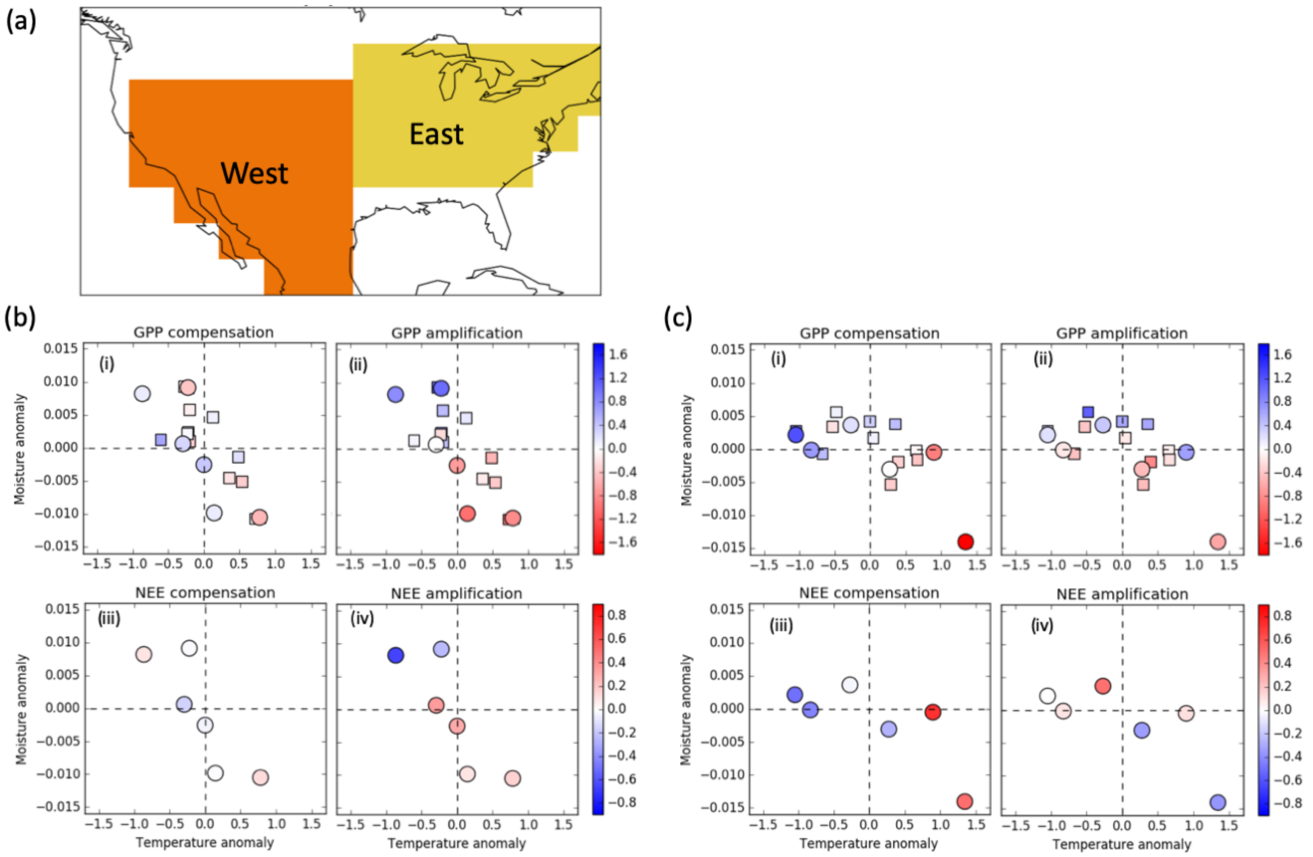

Figure S6. Regional seasonal compensation and amplification components as a function of ΔT and ΔM. (a) Spatial extent of western (orange) and eastern (yellow) regions of North America. (b) Western and (c) Eastern scatter plots of (i) ΔGPP_{comp}, (ii) ΔGPP_{amp}, (iii) ΔNEE_{comp} and (iv) ΔNEE_{amp} as a functions of ΔT and ΔM. NEE covers the period 2010–2015 and GPP covers the period 2001–2017, with circles indication points over 2010–2015 and squares indicating points outside this time period. Colorbars have units of $\mathrm{PgC\,yr^{-1}}$.

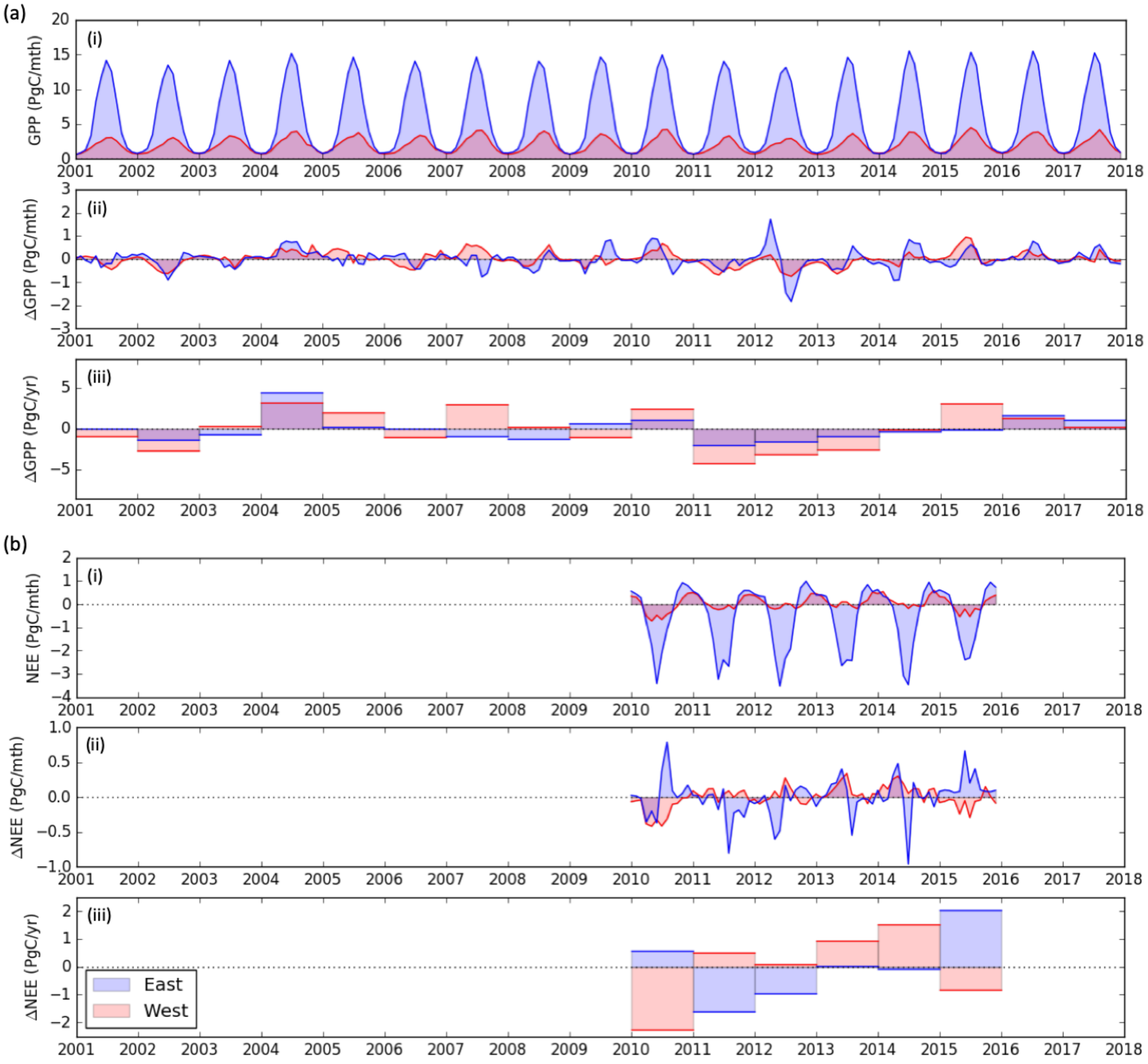

Figure S7. Timeseries of (a) GPP (2001–2017) and (b) NEE (2010–2015) in western (shaded red area) and eastern (shaded blue area) North America. For GPP and NEE, panel (i) shows the seasonal cycle, (ii) shows the monthly anomalies, and (iii) shows the yearly anomalies.

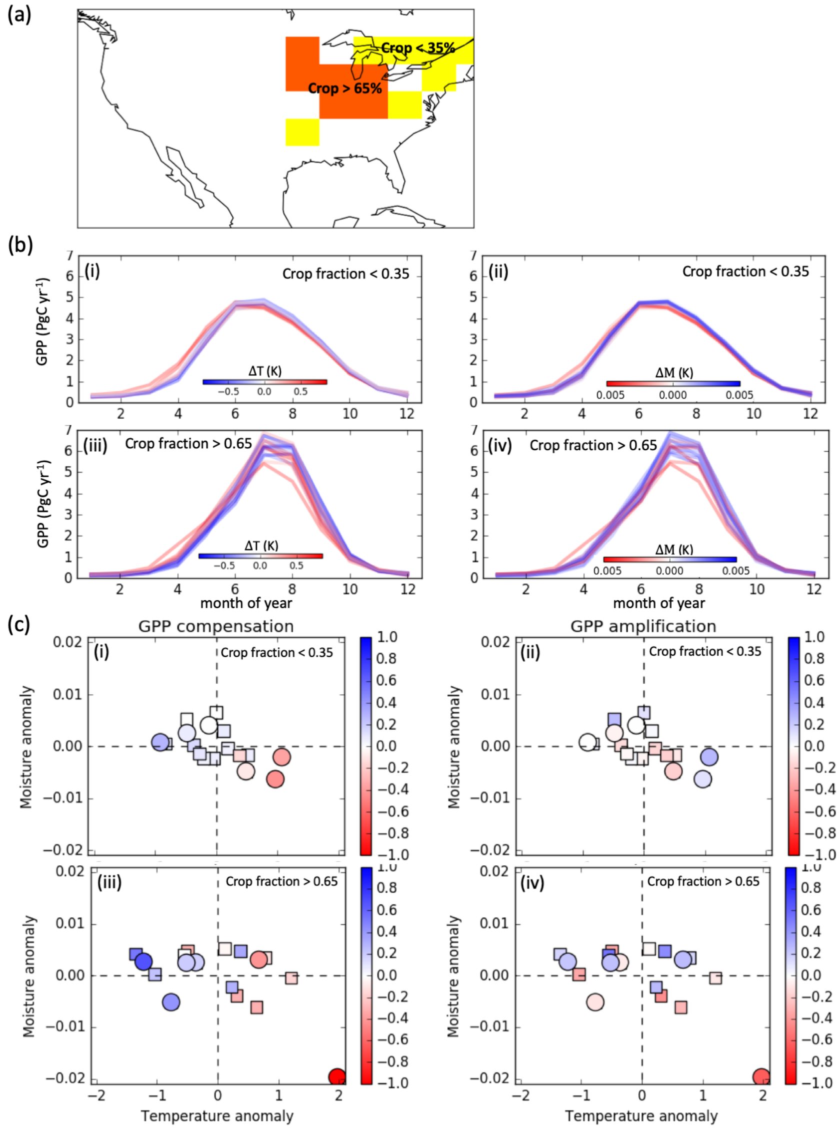

Figure S8. (a) Grid cells with crop fractions > 65% or < 35%. (b) Time series of 2001-2017 GPP as a function of month of year for grid cells with crop fractions < 35% ((i) and (ii)) and crop fractions > 65% ((iii) and (iv)). Curves are colored by Apr-Sep temperature anomaly for (i) and (iii), and are colored by Apr-Sep moisture anomaly for (ii) and (iv). (c) Seasonal compensation ((i) and (iii)) and amplification ((ii) and (iv)) components for grid cells with crop fractions < 35% and > 65%. Note that the outlier for crop fractions > 65% is due to the 2012 North American Drought. Colorbars have units of PgC yr^{-1}.
